# Promotion and prevention regulatory focus LIWC dictionary. Polish adaptation and validation

**DOI:** 10.1371/journal.pone.0288726

**Published:** 2023-07-20

**Authors:** Magdalena Marszałek, Amadeusz Miązek, Marta Roczniewska

**Affiliations:** 1 Institute of Psychology, SWPS University of Social Sciences and Humanities, Warsaw, Poland; 2 Department of International Finance, Poznań University of Economics and Business, Poznań, Poland; 3 Department of Learning, Informatics, Karolinska Institutet, Management and Ethics, Stockholm, Sweden; University of Glasgow, UNITED KINGDOM

## Abstract

This article describes the adaptation and validation of a Polish version of the regulatory focus (RF) Linguistic Inquiry and Word Count (LIWC) dictionary. RF theory proposes that there are two types of self-regulation: promotion (focus on gains, growth, and ideals) and prevention (focus on losses, security, and oughts). Apart from self-report questionnaires, one method to measure RF includes a linguistic analysis. LIWC counts the frequency of words from relevant categories and presents the output as a percentage of all words used in a writing sample. RF LIWC contains two categories: promotion (e.g., achieve, ideal) and prevention (e.g., afraid, fail). To test the psychometric properties of our Polish adaptation of the RF LIWC instrument, we performed three studies. In Study 1 (*N* = 10), experts in RF theory rated the extent to which each dictionary entry was related to promotion and prevention foci. Results showed that words from the promotion category were rated as more promotion than prevention-related, and the pattern was reversed for words from the prevention category. In Study 2 (*N* = 130) we examined the divergent validity of the instrument by experimentally manipulating RF and testing the writing patterns. When a promotion focus was activated, individuals wrote more words from the promotion than prevention category, and the pattern was reversed in the prevention group. Study 3 (*N* = 414) investigated whether the promotion and prevention scores obtained through RF LIWC are linked with results obtained using a self-report questionnaire that measures chronic RF. Promotion scores from RF LIWC correlated positively with chronic promotion RF and prevention scores from RF LIWC correlated positively with chronic prevention RF. These preliminary findings provide initial support for the validity of the Polish adaptation of the RF LIWC.

## Introduction

Psychological research has shown that language expressions indicate mental processes [1]. Written and spoken words used in daily life reflect what people focus on—their thoughts and feelings [2]. For instance, Leis and colleagues [3] showed that people suffering from depression wrote about anger and sadness more frequently than non-depressed individuals. In another study related to extremism, text from Twitter was analyzed [4]; the results showed that extremists’ tweets presented less positive emotion and more negative emotion than partisan users. Further, Pietraszkiewicz and colleagues [5] revealed that descriptions of male-dominated professions in job advertisements contained more words related to agency than ads for female-dominated professions, and the latter consisted of more words related to communion. Finally, through textual analysis of StackOverflow (a social platform for coding questions and answers for IT developers) posts, Bazelli and colleagues [6] found that the top reputed authors were more extroverted persons compared to low reputed authors. Overall, by exploring the way people write, researchers can study temporal states as well as stable individual differences in preferences, perceptions, personality, and motivation [7–9].

Past research [10] indicates that language analysis can aid in studying motivational orientations as they are described by Regulatory Focus (RF) theory [11]. According to RF theory, there are two types of self-regulation: promotion and prevention. While the promotion focus originates from the need for growth and development, the prevention focus stems from the need for safety and security. Promotion-focused persons strive to maximize gains and they tend to engage in risky behaviors. In turn, prevention-focused persons avoid losses, and their behaviors are cautious and based on minimizing errors. In recent years, an interdisciplinary research stream employing textual analysis constructed a Linguistic Inquiry and Word Count (LIWC) dictionary to capture people’s RF (e.g., [10,12,13]). RF LIWC uses dictionaries comprising promotion and prevention words and allows the measurement of constructs by how frequently words from these two categories are used in the text. Past research has successfully employed these dictionaries to study the relationship between RF and gun ownership [12] or RF and responses to restricted stock awards by CEOs [14]. RF LIWC was used to test the relationship between RF and CEO responses to mortality salience [15] as well.

Such text analysis allows for unobtrusiveness in the measurement of motivational concerns. It has a higher reliability and replicability compared to interviews or surveys since it focuses on the words that are directly and objectively visible, observable, and countable [16,17]. Moreover, it can be applied to studies where respondents are difficult to reach, such as company CEOs [18,19].

Despite the importance of studies using the RF LIWC dictionary, the contribution of this tool is limited as it cannot be employed for studies in language contexts other than English without prior translation and validation. Our analysis of articles citing the article introducing RF LIWC [10] has revealed that only one study by Zhang and colleagues [20] conducted such a translation and validation for the Chinese language. Yet, testing the predictions in other language contexts is relevant because many publications make suggestions to use this method in various geographical contexts (e.g., [14,21–23]) which would not be possible in many countries with an original dictionary developed and validated in English. While the literature points to the use of the RF LIWC dictionary in studies of various fields—mostly from psychology (e.g. [12]), and economics or management (e.g. [14,21])—none of them provided a RF LIWC dictionary in Polish. This prevents applications of this tool in this language context. Thus, the overall goal of this project is to validate the RF LIWC dictionary for a lexical analysis of Polish texts.

Our project has four contributions. First, we provide an instrument that enables texts obtained from different sources to be analyzed in an unobtrusive and non-declarative way. Specifically, RF LIWC dictionary allows RF and its consequences to be measured among samples that are difficult to access. For instance, it makes it possible to measure top managers’ RF by applying a content analysis of their written texts like annual reports or shareholder letters [24] and even study different countries’ leaders by analyzing their social media content (e.g., Twitter). Second, we cross the geographic and cultural boundary of the original dictionary by validating this instrument in Polish, which is in line with the recommendation by many publications to employ and test this measurement tool in various geographical contexts (e.g., [14,21–23]. Third, we go beyond the current domains where the RF LIWC dictionary were used in past: usually applied settings of management (e.g., [23,25–28]) Namely, we validated this instrument by analyzing texts related to a more universal topic—where participants described topics of importance in their everyday life. Finally, the RF LIWC dictionary can be used as a manipulation control in an experimental study. Namely, experimental manipulations of RF are often based on participants writing short texts as a response to instructions activating a promotion vs. prevention focus. For instance, participants may be asked to write about their ideals (activation of a promotion focus) or oughts (activation of a prevention focus; see [29]). The RF LIWC dictionary would then enable the effectiveness of a manipulation to be checked without the involvement of, e.g., competent judges, who would have to be trained in theory and make qualitative judgments of each text independently from each other. Thus, this method can provide a cost- and time-effective alternative.

## Regulatory focus theory

RF theory [11,30] was developed to extend the knowledge of people’s approach-avoidance motivation beyond the hedonic principle. It explains how differences in performance, emotions or decision making in approaching pleasure and avoiding pain are influenced by two independent foci (promotion and prevention). In contrast to cognitive prospect theory [31], motivational RF theory [11] provides a comprehensive theoretical framework for understanding an individual’s subjective outcome valuation [32,33]. The motivational process in RF theory involves goal striving instead of goal setting [34]. When striving for a goal, people are concerned with their representation of desired outcomes and undesired outcomes [35,36]. In other words, an individual’s goals affect the psychological meaning and significance of the current value state (the domains of gains and losses), the choice sets (perceived available options), and their dynamic interaction [36].

The starting point of RF theory was the self-discrepancy theory, with its distinction between the "ideal-” and "ought” self [37], otherwise known as standards [38]. Under the “ideal” self-guide, a person represents outcomes as hopes, wishes, and aspirations. Under the “ought” self-guide, a person is concerned with duties, obligations, and responsibilities [11,39]. People have both self-guides, but their predominance vary in two regulatory foci. The “ideal” self-guide is characteristic of a promotion focus and the “ought” self-guide is related to a prevention focus [11]. Under these two self-regulation modes, people are motivated to achieve a certain desired end state. Under a promotion focus, goals are pursued by seeking gains—moving from the current unsatisfactory status quo “0” towards a better state “+1”. In turn, under a prevention focus, goals are pursued by avoiding losses—maintaining the current satisfactory status quo “0” with actions against a worse state “−1” [32]. Self-regulation can, therefore, be described as a characteristic that controls the motivational process of goal pursuit within the framework of accepted standards [38].

RF can be either chronic or situationally cued [11,40]. Chronic self-regulations stems from socialization during childhood and are orthogonal, i.e., do not form two endpoints of a continuum [41] but rather coexists in the person simultaneously on three levels of motivational abstraction: systemic, strategic and tactical. The systemic level reflects a preference for end states, whereas strategic reflects a general preference for means to pursue a goal, and tactical represents a preference for means specific to a given situation [42]. On the systemic level, the two kinds of desired end states can be either a promotion focus on aspirations and accomplishments, or a prevention focus on responsibilities and safety [11]. The two systems of promotion and prevention foci result in differential preferences in goal pursuit strategies [32], i.e., eagerness for a promotion focus vs. vigilance for a prevention focus [36]. The eager strategy emphasizes speed and achieving maximum results [43,44]. In contrast, the vigilant strategy is focused on accuracy and meeting minimum performance standards [43,44]. Because of the negative orientation on outcome, the strategic inclination of people with a predominant chronic prevention focus is to avoid the threat of losing security in the status quo [45]. An individual has both systems, but in a certain moment the concerns of one override the other due to higher chronic accessibility, guiding decision making towards a certain end state [36]. However, on the tactical level, RF can be situationally activated [11].

People take on multiple tactics (otherwise known as means) in their goal pursuit, but multiple goals can also be addressed with the same tactic. This is because goals and means are different levels of a motivational system [36]. The ability to change tactics in response to momentary stimuli is possible due to the independence of each level of motivational abstraction [35]. Their independence involves multifinality (the same means may serve multiple goals), and equifinality (the same goal may be served by more than one means). Compared to the higher, systemic and strategic level goals involving the decision maker’s motivation, the lower, tactical level goals involve the choice of a more or a less risky option [36]. Chronic RF can be situationally displaced by a promotion focus in gain/non-gain circumstances or a prevention focus in loss/non-loss circumstances [46,47]. Momentary situations are capable of triggering either promotion or prevention focus self-regulatory concerns [41]. Satisfaction with one’s goal at or above the reference point in the domain of gains does not equal a preference for choosing the less risky option, and dissatisfaction with one’s goal below the reference point in the domain of losses does not equal a preference for choosing the riskier option [33].

There are several measurement tools that allow one to study chronic dispositions in RF. Most of them are self-reports, such as the Regulatory Focus Questionnaire provided by Higgins and colleagues [48], the Regulatory Focus Scale proposed by Fellner [49] or the Regulatory Focus measurement by Lockwood and Kunda [50]. The Regulatory Focus Strength Measure, on the other hand, allows researchers to study RF based on response times of listing their ideal vs. ought attributes [51]. While the self-report measures have many advantages like efficiency in collecting data and that they are relatively inexpensive, as well as enabling data concerning many variables in a short period of time [52]—they have some limitations as well. For example, self-report instruments can introduce artifactual variance in research by susceptibility to social desirability bias [53]; they are also not applicable when the participants are difficult to reach. While another type of tool, the Regulatory Focus Strength Measure [51], may be considered free from social desirability in part because it is based on response times, its drawback is that that it may require a presence in the lab, reliable software to measure reaction times, and it may be considered intrusive. Linguistic analysis seems to be resistant to the above-mentioned limitations.

## Text analysis using LIWC

As exemplified earlier, text analysis allows researchers to investigate people’s preferences [9], affect [54], regulatory mode (locomotion vs. assessment [55,56]), temporal focus (a past, present, and future focus [57]), cognitive complexity (differentiation and nuance in thinking [58]) and many other psychological constructs and states. For instance, text analysis makes it possible to recognize signs of depression [3]. A commonly used language analysis technique revealing individuals’ cognitive schemas in their spoken or written material is content analysis [59]. This method is placed at the intersection of the qualitative and quantitative traditions, and focuses on the identification of both manifested (easily observable meanings in a body of text), and latent content (underlying meanings of texts) [60]. One of the most frequently used tools for content analysis [61] that allows researchers to analyze language patterns and their co-occurring constructs is Linguistic Inquiry and Word Count (LIWC). LIWC uses dictionaries in a specific type of content analysis that measures constructs by how frequently words are used in the text [59]. LIWC includes built-in dictionaries that can be used to analyze texts in several languages, as well as the option to upload custom-made dictionaries [10,59].

Researchers can create their own dictionaries that reflect any psychological constructs of their choice [10,59]. There have been attempts to capture RF in writing samples indirectly by, e.g., studying emotional patterns related to promotion (dejection–elation) and prevention (agitation–quiescence) to examine motivational and language-driven processes in an internet support group for those who quit smoking [62]. However, this study measured only the emotional dimensions of the RF. The seminal work necessary to study RF in humans was conducted by Gamache and colleagues [10], who developed and validated the RF LIWC dictionary in several steps. First, they created two lists of words indicative of a promotion (e.g., achieve, ideal, speed) vs. prevention (afraid, fail, safety) focus based on earlier literature on RF, survey measures of RF, and word fragment completion tests [10]. Second, to verify the content validity of these lists, they asked 25 experts (researchers in organizational science with publications on regulatory focus) to code each word as reflecting either a promotion focus or prevention focus [10]. In a third step, to test convergent and discriminant validity, the researchers used RF LIWC to analyze writing samples and correlated them with survey-based measures of regulatory focus and personality traits (e.g., Five Personality traits, and affectivity [10]). The results demonstrated the expected relationships between self-report scores and LIWC output for RF, supporting both convergent validity and discriminant validity [10]. Finally, for predictive validity, they successfully tested theoretically derived relationships between Chief Executive Officers’ (CEO) promotion focus and their firms’ acquisition activity, while the relationship was opposite for CEOs’ prevention focus [10]. The resulting RF LIWC dictionary has been adopted by other researchers. For example, RF LIWC dictionary was applied to investigate how RF predicts consumer behavior in response to service bundles (e.g., theater season-tickets, vacation packages, or annual sports passes) [63]. Pham and colleagues examined RF fit and regulatory mode fit through text analyses of data from 10,547 music app consumers, and they found that that regulatory focus-mode fit (vs. non-fit) decreases variety-seeking [56].

## Overview of scale adaptation procedure

Our project aimed to adapt and validate the RF LIWC dictionary to obtain a Polish version. To achieve this goal, we conducted three studies with diverse samples and procedures. In the first step, to acquire the final list of words from promotion and prevention categories and to test the internal validity of this dictionary, we conducted a study with competent judges (*N* = 10). Next, to test the theoretical validity of the RF LIWC dictionary, we conducted an experimental study (Study 2; *N* = 130). Study 2 investigates whether the Polish adaptation of RF LIWC produces the expected differences in writing patterns as a result of activated foci (promotion or prevention). In this study, we manipulated promotion and prevention self-regulation and we measured the frequency of using words from promotion and prevention categories. Finally, to test whether the Polish adaptation of the RF LIWC dictionary enables the detection of differences in the writing patterns derived from chronic self-regulation, we conducted a correlational study (Study 3). To test the relationship between chronic RF and the frequency of using words from promotion or prevention categories, we used the Promotion and Prevention Self-Regulation Scale [38].

## Study 1

The aim of Study 1 was to (a) translate the original RF LIWC dictionary to Polish and (b) test the internal validity of the Polish RF LIWC dictionary. For this purpose, experts in RF theory were asked to rate the words included in the promotion and prevention categories within the RF LIWC dictionary on two separate scales: the extent to which they are related to promotion and the extent to which they are related to prevention. We assumed that the ratings of promotion and prevention by the experts would be congruent with the category of the words. More specifically, we hypothesized that:

Hypothesis 1 (H1). Words from the promotion category in the RF LIWC dictionary will be rated as more related to a promotion than a prevention focus.
H2. Words from the prevention category in the RF LIWC dictionary will be rated as more related to a prevention than a promotion focus.
H3. Words from the promotion category in the RF LIWC dictionary will be rated as more related to promotion foci than words from the prevention category.
H4. Words from the prevention category in the RF LIWC dictionary will be rated as more related to prevention foci than words from the promotion category.

The data that support the findings of this study are openly available in the OSF: https://osf.io/kz3x4/?view_only=1bfc918ab953478b9a7b1a15051d8ece.

### Methods

#### Participants and procedure

Polish experts in RF theory were identified based on the authorship of research articles about RF. We invited 16 experts via e-mail to participate in an online anonymous study and received 10 completed surveys. We did not collect any personal or metric data to allow for anonymous entries. The aim of the study and the informed consent form were presented on the first page of the survey. Next, the participants were asked to rate on two separate scales the extent to which each word was related to promotion and the extent to which each word was related to prevention, using 5-point response scales ranging from 1 (*definitely NOT promotion- / prevention-related*) to 5 (*definitely promotion- / prevention-related*). The definitions of both constructs were presented at the top of the page. At the end of the survey, participants could share any additional thoughts and comments about the list.

#### Materials

To obtain the Polish version of the RF LIWC dictionary, we performed the following steps. In the first step, one of the authors translated the list of Regulatory Focus Words (RFW [10]) to Polish using 3 different English-Polish dictionaries [64–66]. All possible translations provided by these dictionaries were written down. In the second step, two other authors of this manuscript chose the best translation per entry independently from each other. Our basic rule was that for one entry in the original dictionary, one Polish equivalent would be assigned. We made this rule for two reasons. First, we assumed that as it was an adaptation of a validated RF LIWC dictionary, we should keep as many similarities as possible between the original dictionary and its translation, including the number of words in the original RF LIWC dictionary and its Polish version. Second, we believe that it is crucial to create a dictionary that is as effective and short as possible. However, we allowed for exceptions to the above-mentioned rule in justified cases. For example, the word “fail” in the English language has more meanings than its Polish equivalent; therefore, for the word “fail” we assigned three Polish words instead of one (Polish equivalents for word “fail”: “błąd”, “porażka”, “mylić”). On the other hand, for the words “duty” and “obligation”, we found only one suitable Polish equivalent (“obowiązek”). Four words did not receive Polish equivalents: “hoping”, “escaping”, “security”, and “obligation”. Additionally, Polish questionnaires measuring RF were also taken into consideration when the words were translated (The Promotion and Prevention Self-Regulation Scale [38]; Regulatory Focus Questionnaire [48]) to make sure that we did not omit any Polish phrases that are crucial from the RF theory perspective. As a result of this process, the following words were added: “doskonalić” (“improve”), “marzenie” (“desire”), “mylić” (“fail”), “porażka” (“fail”), “sprawdzać” (“verify”), “uważność” (“careful”), “wyzwanie” (“challenge”). After making independent decisions about each translation, the two experts met and agreed upon the final list of words. The final list comprised 29 promotion and 26 prevention words (see S1 Table). In the third step, to obtain input to the LIWC engine all grammatical forms and variants (including conjugations and declensions) for these words were generated. This procedure was necessary because Polish is an inflectional language where, e.g., the verb is conjugated depending on tense, aspect, mood, person, number, and grammatical gender. For this purpose, we used two Polish dictionaries: the Great Dictionary of the Polish Language (WSJP [67]) and the Dictionary of the Polish Language (PWN [68]).

### Results

#### Analysis strategy

To test the level of concordance in the ratings of competent judges, we calculated the Intraclass Correlation Coefficient (ICC). Next, we employed a paired-samples t-test (see S2 Table) to test whether the differences between the promotion vs. prevention ratings for each word separately are significant and in line with the words’ category. Finally, we performed a repeated measures ANOVA, to test Hypotheses 1–4 describing the interaction effect between the type of word and experts’ rating. The design was 2 (category of words: promotion, prevention) x 2 (rating the words: promotion-related, prevention-related). Pairwise analyses were conducted using the Bonferroni test.

#### Preliminary analyses

ICC estimates (95% CI) were calculated using SPSS Statistics version 27 based on a mean-rating (k = 10), absolute-agreement, two-way mixed-effects model. The ICC for average measures was .97 (95% CI = .96–.98, p < .001), which means that the experts were highly similar in their ratings [69]. Pairwise comparisons (see S2 Table) showed that for only four words (two from promotion and two from prevention categories) were insignificant differences between their promotion and prevention ratings with Cohen’s d below 0.70. These words were excluded from the list. The excluded words were as follows: “zarabiać” (“earn”), “ryzyko” (“risk”), “spieszyć” (“speed”), “uważność” (“vigilance”). We also excluded the word “chcieć” (“wish”). The word “wish” was translated into the Polish word “chcieć”; however, its role and meaning in communication is much closer to the English word “want”. As a consequence, in the Polish language, this word can equally express the desired state for promotion and prevention foci. This was reflected in experts’ ratings. Finally, we ended up with 26 words in the promotion category and 24 words in the prevention category in the Polish RF LIWC dictionary (see S1 Table).

#### Hypotheses testing

**Fig 1:**
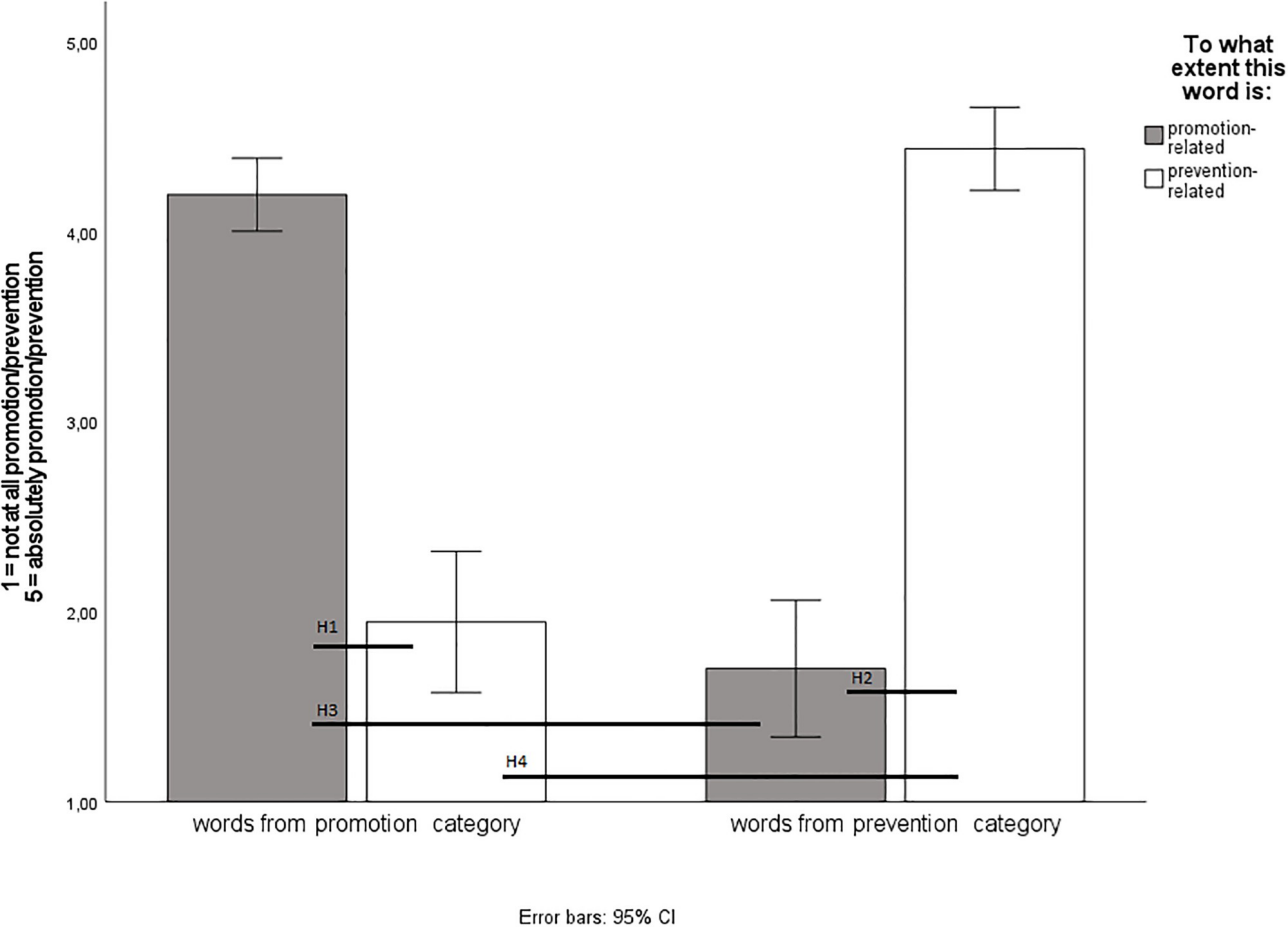
Promotion- and prevention-related ratings made by competent judges. Study 1. Note. *N* = 10.

To investigate Hypotheses 1–4, we employed a repeated measures ANOVA. As expected, the analysis showed a significant interaction effect between the category of a word and experts’ rating, F (1, 9) = 149.912, p < .001, η_p_^2^ = .94. The results are presented in Fig 1.

The pairwise analyses supported Hypothesis 1: the words included in the promotion category in the RF LIWC dictionary were rated as more related to a promotion focus (*M* = 4.19 *SD* = 0.27, *p* < .001) than to a prevention focus (*M* = 1.95, *SD* = 0.52). Our findings also supported Hypothesis 2: the words included in the prevention category in the RF LIWC dictionary were rated as more related to a prevention (*M* = 4.44, *SD* = 0.31, *p* < .001) than to a promotion focus (*M* = 1.70, *SD* = 0.50). In line with Hypothesis 3, the words included in the promotion category in the RF LIWC dictionary were rated as related to promotion foci (*M* = 4.19, *p* < .001) more than words included in the prevention category (*M* = 1.70). Finally, our finding also supported Hypothesis 4: words included in the prevention category in the RF LIWC dictionary were rated as related to prevention foci (*M* = 4.44, *p* < .001) more than words included in the promotion category (*M* = 1.95).

To summarize, we observed high agreement between the experts in their ratings of the words. We obtained a significant interaction between the category and ratings, which demonstrates that words included in the Polish RF LIWC dictionary are correctly assigned to the promotion vs. prevention category. Overall, we believe that this preliminary version of the Polish RF LIWC dictionary has good content validity.

## Study 2

In Study 2, we tested the theoretical validity of the Polish version of the RF LIWC dictionary. We focused on divergent validity, understood as “*the ability of a measure to produce relevant group differences”* [70]. Here, the group differences relate to the frequency of promotion and prevention words used as a function of specific self-regulation. Thus, the RF LIWC dictionary should produce a high promotion and a low prevention score when promotion self-regulation is activated (H5). Conversely, the instrument should yield a high prevention and a low promotion score when prevention is activated (H6). Moreover, the RF LIWC dictionary should provide a higher promotion score when promotion self-regulation is activated than when prevention self-regulation is activated (H7). Finally, the RF LIWC dictionary should yield a higher prevention score when prevention self-regulation is activated than when promotion self-regulation is activated (H8).

Therefore, we performed an experimental study, where regulatory foci were activated using an experimental manipulation, and we analyzed texts produced by the participants as a reaction to our prompt. Our dependent variables were the frequencies of words from promotion and from prevention categories.

The study was pre-registered (https://aspredicted.org/DM6_BCQ). The data that support the findings of this study are openly available in the OSF: https://osf.io/kz3x4/?view_only=1bfc918ab953478b9a7b1a15051d8ece.

### Method

The Ethics Review Board at SWPS University of Social Science and Humanities, Poland; Faculty of Psychology in Sopot concluded that there is no risk of harm related to participation in this study (decision no. WKE/S2022/16/II/112). Each participant gave written informed consent before participating in this study.

### Power analysis

The G*Power calculator [71] was used to estimate the required number of participants for a mixed ANOVA within-between interaction with 80% power and alpha of .05. The design was 2 (activated focus: promotion self-regulation, prevention self-regulation; between-subject factor) x 2 (the frequency of words used from a particular category: promotion, prevention; within-subject factor). We have no previous results to rely on with regard to what effect sizes we should predict for these particular comparisons, hence we conservatively assumed that our effect of interest would probably be small-to-medium and f was set to 0.20. The correlation among repeated measures was set as −.10 [10]. A priori sample size calculation estimated a sample size of 110. We expected that some participants may not adhere to the task that introduced the manipulation, so we aimed to recruit at least 130 subjects. For more details regarding exclusion criteria, see also the pre-registration file: https://aspredicted.org/DM6_BCQ.

#### Participants

The participants were recruited through an internal university database. Participants took part in the study for required credit. Based on exclusion criteria (see pre-registration file: https://aspredicted.org/DM6_BCQ), we excluded 3 records of participants who completed the survey in less than 7 minutes. The final study sample (*N* = 130) consisted of 112 (87%) women and 17 men (one person did not provide information about their gender), aged between 19 and 51 (*M*_*age*_ = 29, *SD* = 9.28).

#### Materials and measures

*Manipulation of self-regulation*. The respondents were asked to carefully read the instructions and to describe (a) how their current hopes and aspirations are different now from when they were growing up (activation of a promotion focus) or (b) how their current duties and obligations are different now from when they were growing up (activation of a prevention focus). This manipulation was based on previous research [29,39]. Participants were instructed to write their response using c. 10 sentences (for similar requirements, see Gamache et al. [10]).

*Frequency of promotion vs*. *prevention words*. To compute the frequency of using words from the promotion and prevention categories, we used LIWC engine with the Polish version of the RF LIWC dictionary described above. The dependent variables were log-transformed before the analyses to remove skewness—the frequency of words has been shown to be right-skewed in past research (e.g., [10]), as well as in our pilot studies.

#### Procedure

The study was conducted online. The purpose of the research and respondents’ rights were presented on the first page of the survey. Then, a randomization procedure (a standard procedure implemented in Qualtrics) assigned participants to one of two conditions: with a manipulation activating promotion self-regulation (*N* = 68) or prevention self-regulation (*N* = 62). In the promotion condition, the participants described the differences between their present and past hopes and aspirations, while in the prevention condition they described their duties and obligations (see S1 Appendix).

### Result

#### Analysis strategy

To test Hypotheses 5–8, describing the interaction effect between the activation of a promotion vs. prevention focus and the frequency of writing words from the promotion vs. prevention category, we performed a mixed ANOVA. Additionally, to investigate effect sizes in pairwise comparisons, we calculated Cohen’s *d*, using Effect Size Calculator [72].

#### Hypotheses testing

The text entries prepared by the participants were on average 203.99 words long (*SD* = 35.54).

First, we did not observe a main effect of frequency of using promotion (*M* = 1.83) vs. prevention (*M* = 2.16) words, *F* (1,128) = 0.01, *p* = .940, *η*_*p*_^*2*^ = .009. This means that, overall, participants used the same number of words from the promotion and prevention categories, regardless of the condition. Second, the analysis revealed a main effect of the experimental condition, *F* (1, 128) = 25.71, *p* < .001, *η*_*p*_^*2*^ = .17). Regardless of the category of words (promotion/prevention), individuals in the promotion condition wrote fewer words (*M* = 1.61; p < .001) than individuals in the prevention condition (*M* = 2.38).

**Fig 2:**
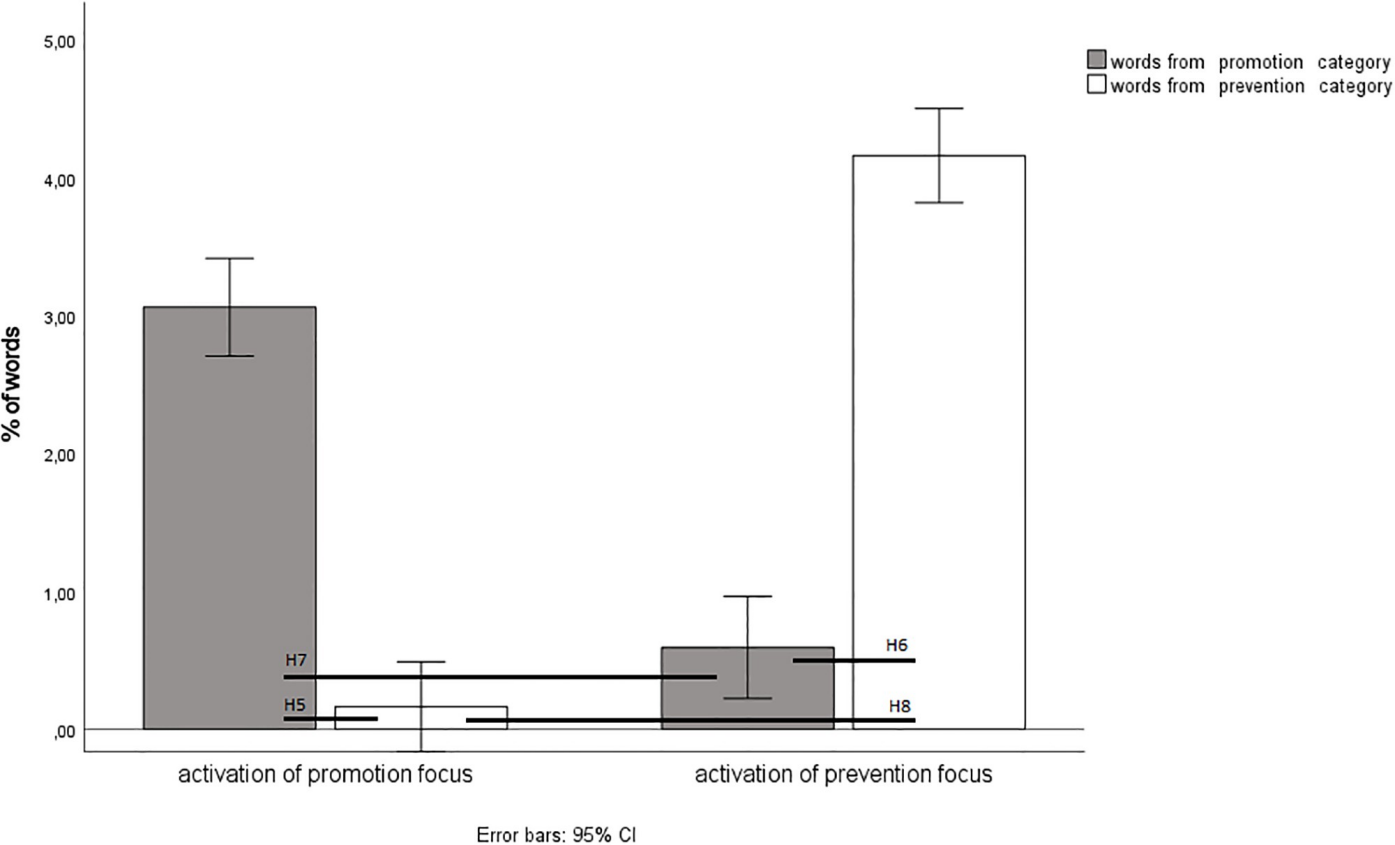
Frequency of using words from the promotion and prevention categories depending on the activation of a promotion vs. prevention focus. Note. *N* = 130.

As expected, the analysis showed a significant interaction effect between the condition (activated promotion vs. activated prevention self-regulation) and the frequency of using words from the promotion vs. prevention category, *F* (1,128) = 720.51, *p* < .001, *η*_*p*_^*2*^ = .85). The results are presented in Fig 2.

The pairwise analyses supported Hypothesis 5: activation of promotion RF led to words from the promotion category (promotion words) being used more frequently than words from the prevention category (prevention words) (*M* = 3.06.90, *SD* = 1.91 vs *M* = 0.16, SD = 0.33, *p* < .001, *d* = 2.59). Our findings also supported Hypothesis 6: activation of prevention RF resulted in prevention words being used more frequently than promotion words (*M* = 4.16, *SD* = 1.94 vs *M* = 0.59, *SD* = 0.73, *p* < .001, *d* = 2.67). In line with Hypothesis 7, activation of promotion RF led to more frequent use of promotion words than activation of prevention RF (*M* = 3.06 vs *M* = 0.59, *p* < .001, *d* = 1.87). Finally, our finding also supported Hypothesis 8: activation of prevention RF led to more frequent use of prevention words than activation of promotion RF (*M* = 4.16 vs *M* = 0.16, *p* < .001, *d* = 3.52).

#### Additional analyses

In pre-registration, we included information about an additional sensitivity analysis. We excluded from the analyses those participants who did not use any words from either the promotion or prevention categories (i.e., had scores of 0 in both promotion and prevention results from LIWC output). There were two persons (1.5%) who obtained scores of 0 in both promotion and prevention results from LIWC output, and the re-analyzed findings did not differ from those reported above.

To summarize, we found support for the influence of manipulated promotion and prevention RF on the frequency of using promotion and prevention words in a writing sample produced as a response to a prompt. The observed differences were large and in the expected direction, which supports the divergent validity of the instrument.

## Study 3

Overall, Study 2 supported our hypotheses concerning the differential writing patterns in terms of promotion and prevention vocabulary, depending on activated RF. However, while the experimental design of this study allows us to make causal claims about the influence of prompted self-regulation on the frequency of using promotion or prevention words, Study 2 does not allow us to conclude that RF LIWC is able to detect differences in the writing patterns that stem from chronic self-regulation. Yet, this is important to test whether the Polish version of RF LIWC can become an implicit measure of RF to potentially be able to substitute questionnaires measuring RF in cases where it is needed. Therefore, we performed a correlational study to test the following hypotheses:

H9. The higher the self-reported chronic promotion self-regulatory focus (but not the prevention), the more frequent the use of words from the promotion category in the RF LIWC dictionary.
H10. The higher the self-reported chronic prevention self-regulatory focus (but not the promotion), the more frequent the use of words from prevention category in the RF LIWC dictionary.

**Fig 3:**
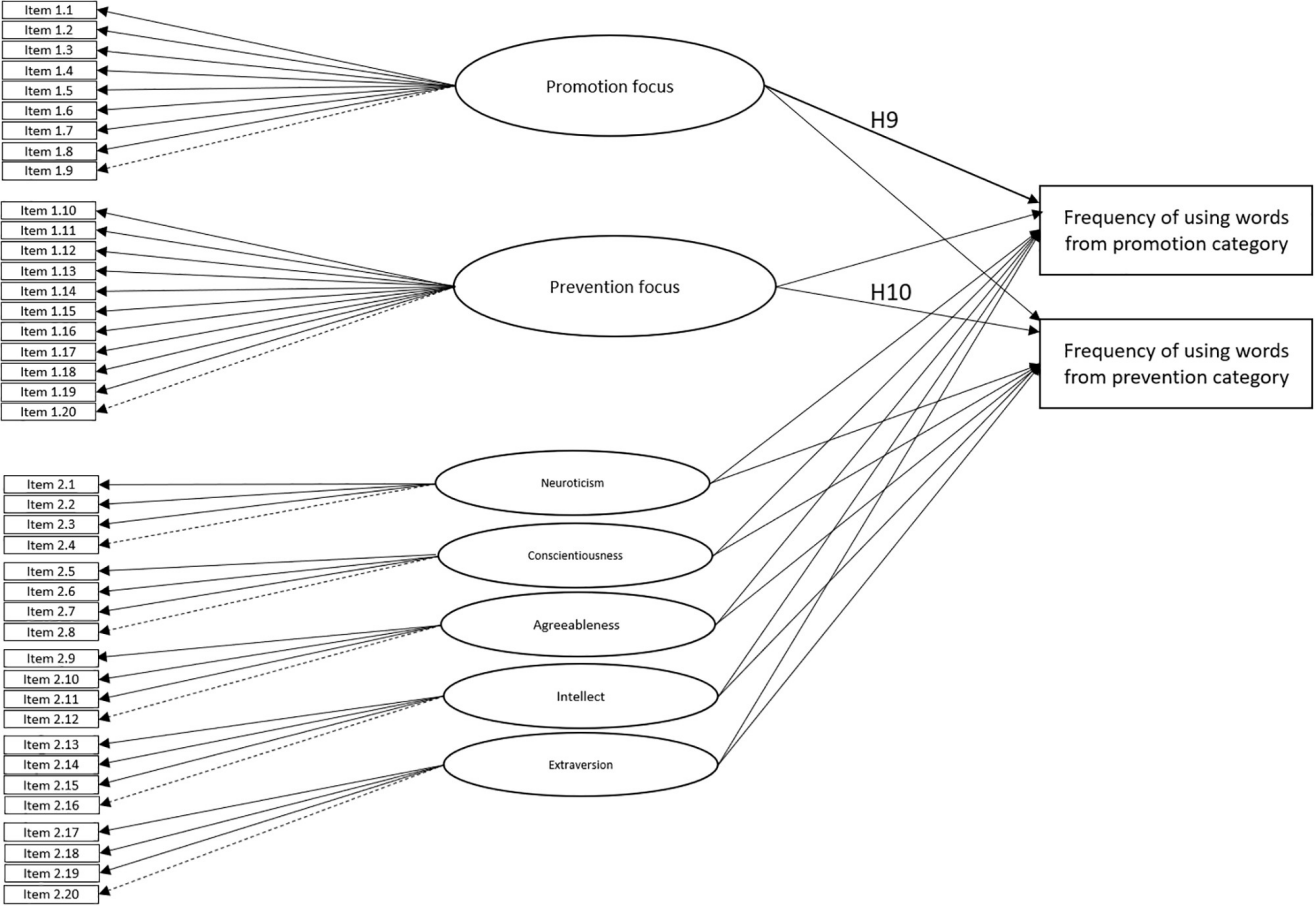
Conceptual model in Study 3.

Additionally, similarly to Gamache et al. [10], in Study 3 we measured Big Five personality to control the potential influence of personality traits on the links between chronic RF and writing patterns [10]. Based on previous literature (i.e., [34,73–75]), Gamache and colleagues [10] pointed out that while self-regulation has a direct impact on one’s behavior, personality traits influence rather indirectly via motivational processes. Thus, we decided to control the impact of personality by using the BIG Five model (see Fig 3).

The study was pre-registered (https://aspredicted.org/MR4_73D). The data that support the findings of this study are openly available in the OSF: https://osf.io/kz3x4/?view_only=1bfc918ab953478b9a7b1a15051d8ece.

### Method

The Ethics Review Board at SWPS University of Social Science and Humanities, Poland; Faculty of Psychology in Sopot concluded that there is no risk of harm related to participation in this study (decision no. WKE/S2022/16/II/112). Each participant gave written informed consent before participating in this study.

#### Power analysis

The G*Power calculator [71] was used to calculate the required number of participants for a linear multiple regression (fixed model, *R*^*2*^ increase) for seven predictors (2 regulatory foci, 5 personality traits from the Big Five model). We expected a small effect size, thus we set f^2^ to .025. The minimum sample size was estimated at 389. Because we anticipated excluding some participants based on prior pilot studies (see: Participants or pre-registration file: https://aspredicted.org/MR4_73D), we initially recruited over 600 individuals.

#### Participants

The participants were recruited through a professional research agency (Pollster; pollster.pl) who owns a platform that allows individuals to perform online tasks in exchange for points that can be transferred to rewards or money. Based on exclusion criteria (see pre-registration file: https://aspredicted.org/MR4_73D), we excluded 196 records of participants: 16 participants who completed the survey in less than 8 minutes, 61 who did not follow the instructions (e.g., wrote a text not in accordance with the instructions, copied the text from other sources, wrote nonsense, etc.), 56 who indicated contradictory answers (e.g., marked the same answer for the item and its reverse version), and 63 as the measures that were more than 3 times the mean of all Cook’s distances (see S3 Table).

The final study sample (*N* = 414) consisted of 199 (48%) women and 214 men (1 person did not provide information on their gender) aged between 18 and 85 (*M*_*age*_ = 46, *SD* = 16.17).

#### Materials and measures

*Writing instructions*. Because we aimed to obtain writing samples that represent chronic self-regulation, we needed a writing prompt that did not activate a promotion or prevention focus on its own. The initial instruction was based on a study performed by Gamache et al. [10]. To obtain neutral writing instructions, we conducted three pilot studies. Every iteration resulted in changes introduced to the instructions. The changes were related to words that could influence the participants or activate promotion or prevention self-regulation (the content of the trial instructions can be found in the S2 Appendix). In the final version of the instructions, the participants were asked to write a text passage (circa 10 sentences) about important topics in their current life. The instructions asked the participants to think about different current topics/goals/issues in their lives. They were prompted to reflect on what is important to them now and how they attend to it. The mean length of the texts was *M* = 163.92 (*SD* = 38.80) words.

*Frequency of promotion vs*. *prevention words*. To compute the frequency of using words from the promotion and prevention categories, as in Study 2, we used LIWC engine with the Polish version of the RF LIWC. These variables were log-transformed before the analyses to remove skewness—the frequency of words has been shown to be right-skewed in past research (e.g., [10]), as well as in our pilot studies.

*Promotion and prevention self-regulation* was measured using the Promotion and Prevention Self-Regulation Scale (PPSS) [38]. The scale consists of five subscales (promotion standards [5 items], promotion self-control [4 items], prevention standards 4 items], prevention self-control [7 items] and strength of motivation [7 items]; see also PPSS translated into English in S3 Appendix). For the purposes of this study, we used four subscales: promotion standards (e.g., “I dream and make my dreams come true”, *α* = .75), promotion self-control (e.g., “I like new challenges; *α* = .65), prevention standards (e.g., “I usually do what I have to do”, *α* = .60), prevention self-control (e.g., “While completing the task, I focus on making as few mistakes as possible”, *α* = .72). The reliability of promotion RF (a factor comprising both standards and self-control subscales) was *α* = .81. The reliability of prevention RF (a factor comprising both self-control and standards subscales) was *α* = .72. The respondents indicated their agreement with each statement using 5-point scale from 1 (*I completely disagree*) to 5 (*I completely agree*).

*Big five personality traits* were measured using the Short Questionnaire of Big Five—IPIP in its Polish adaptation [76] (20-item scale: 4 items for each dimension). Example items are “I am the life of the party” (extraversion; *α* = .88), “I do not have a good imagination” (intellect [reversed]; *α* = .78), “I sympathize with others’ feelings” (agreeableness; *α* = .67), “I get chores done right away” (conscientiousness; *α* = .78) and “I have frequent mood swings” (neuroticism *α* = .83). Items were rated on a 5-point scale, ranging from 1 (*It is definitely not about me*) to 5 (*It is definitely about me*).

#### Procedure

The purpose of the research and the participants’ rights were presented on the first page of the survey. Next, the participants completed two questionnaires: to measure promotion and prevention self-regulation and five personality traits. In the last step, the participants were asked to write c. ten sentences about important topics in their current life.

### Results

#### Analysis strategy

To investigate the construct validity of the measures and measurement model, we conducted a series of confirmatory factor analyses (CFA) using the Lavaan package [77–79] in R software [80] (see S4 Appendix.) The model is considered to fit the data when the following values are acquired: RMSEA < .08 [81] and TLI and CFI > .90 [82].

To test our hypotheses, we performed structural equation modeling (SEM) using the Lavaan package [77–79] in R software [80]. The frequency of using words from promotion and prevention categories served as two observed variables set as dependent variables. The measurement model contained two latent predictors (questionnaire-based variables: the first variable consisted of 9 promotion-related items and 11 prevention-related items) and five latent covariates (five dimensions of Big Five personality traits: each dimension consisted of 4 items). We included Big Five personality traits in the same measurement model to examine whether self-regulation explained additional variance in the frequency of using words from the promotion vs. prevention category over and above five personality traits.

As in Study 2, the dependent variables were log-transformed before the analyses to remove skewness. To test our hypotheses, we created a model that contained seven latent variables (five predictors: promotion and prevention foci and five personality traits) and two observed variables (dependent variables: frequency of writing words from promotion and prevention categories). However, values of CFI and TLI (see S4 Table) showed that the model did not fit the data well enough. Hence, we added the correlations of residuals of variables indicated by the calculation of modification indices (modification indices > 25; included correlations of residuals for eight variables [83,84]) to the model. After this modification, we obtained better fit indicators (see comparisons of models in S4 Table); hence, we decided to base our findings on the model with better fit. We included correlations of residuals only for items which measured the same variables and when their similar wording justified the occurrence of correlations (items 4 and 11, 4 and 9 for prevention standards; items 18 and 23 for promotion standards; items 1 and 14 for promotion control; items 3 and 13, 2 and 12, and 8 and 18 for the Big Five personality traits). Examples of these items are: (1) “I often pay special attention to what I do so that it is not perceived negatively by others” and “Most often, my actions are consistent with what others expect from me”; (2) “I act and I turn dreams into reality” and “I go for my dreams” (two items from the prevention and two items from the promotion standards dimension) from (PPSS) [38] or (3) “I leave my stuff wherever I go” and “I often forget to put things away” (conscientiousness dimension, reversed items) from IPIP[76].

#### Hypotheses testing

As Table 1 shows, a chronic promotion focus was positively associated with using words from the promotion category (*β* = .29, *p* = .020) while chronic prevention focus was not positively associated with using words from promotion category (*β* = .03 *p* = .754). This pattern provides support for Hypothesis 9. Next, in line with Hypothesis 10, a chronic prevention focus was positively and significantly associated with using words from the prevention category (*β* = .27, *p* = .046); a chronic promotion focus was negatively related with using words from the prevention category (*β* = −.36, *p* = .011). Interestingly, a promotion focus was a negative predictor of using words from the prevention category. As expected, we did not observe any significant relationships using words from the promotion and prevention categories and five personality traits. Our measurement model did not reach a satisfactory fit (CFI = .81, Robust CFI = .82, TLI = .79, Robust TLI = .80, RMSEA = .06, Robust RMSEA = .06 [90% CI: .05―.06]). This means that the results presented by this model should be interpreted with caution.

**Table 1:** Results for alternative SEM models explaining the frequency of promotion and prevention words with and without modification indices in Study 3.

		two-factors regulatory focus (promotion and prevention) with modification based on modification indices + five personality traits				two-factors regulatory focus (promotion and prevention) without modification based on modification indices + five personality traits			
Dependent variable	Predictor	Estimate (*b*)	*SE*	*p*	*β*	Estimate (*b*)	*SE*	*p*	*β*
Frequency of words from promotion category—After Log transformation	Promotion self-regulation	**0.27**	**0,12**	**.020**	**.29**	**0,26**	**0.10**	**.012**	**.27**
	Prevention self-regulation	0.06	0.19	.754	.03	0.09	0.12	.442	.06
	Extraversion	-0.03	0.04	.440	-.06	-0.04	0.04	.355	-.07
	Agreeableness	0.09	0.11	.416	.07	0.124	0.10	.197	.08
	Conscientiousness	-0.07	0.06	.272	-.10	-0.09	0.05	.094	-.11
	Neuroticism	-0.04	0,05	.415	-.07	-0.06	0.05	.268	-.09
	Intellect	-0.08	0,08	.266	-.10	-0.07	0.07	.323	-.08
Frequency of words from prevention category—After Log transformation	Promotion self-regulation	**-0.24**	**0.09**	**.010**	**-.36**	**-0.21**	**0.08**	**.005**	**-.30**
	Prevention self-regulation	**0.40**	**0.20**	**.046**	**.27**	**0.25**	**0.12**	**.040**	**.22**
	Extraversion	0.02	0.03	.507	.05	0.014	0.03	.684	.03
	Agreeableness	-0.03	0.09	.761	-.03	-0.03	0.07	.730	-.02
	Conscientiousness	-0.05	0.05	.345	-.10	-0.07	0.04	.115	-.11
	Neuroticism	-0.07	0.04	.105	-.15	-0.07	0.04	.078	-.15
	Intellect	0.07	0.06	.212	.13	0.07	0.05	.195	.12

Note. *N* = 414.

Additionally, we tested whether we had enough power to detect the expected relationships given our simple size, using software to compute power for RMSEA (Preacher & Coffman [85]). The results demonstrated that with a sample size of 414 and alpha level of .05, we could detect significant relationships between predictors and two dependent variables with power of a 0.82. This means that we had an 18% chance of not detecting relationships that actually exist.

#### Additional analyses

The self-report scale to measure RF that we used in Study 3 divides promotion and prevention foci into standards and self-control, and it has a hierarchical factor structure [38]. However, the sample size did not allow us to perform a hierarchical SEM analysis, as the model did not converge [86]. Thus, for exploratory purposes, we employed a non-hierarchical analysis where the predictors were promotion standards, promotion self-control, prevention standards, prevention self-control, and five personality traits. However, this model did not allow us to make any conclusions due to very high covariances between standards and self-control—promotion standards and promotion self-control (*cov* = .83) as well as prevention standards and prevention self-control (*cov* = .45) [87]. One method to manage over-correlated predictors is to remove one of them from the model or combine these variables into a common factor [87]. To test the relationships of both standards and control for promotion and prevention foci despite these limitations, we decided to create two separate models. The first model investigated relations between promotion and prevention standards (controlling for five personality traits) and frequency of writing words from the promotion and prevention categories. The second model included promotion and prevention self-control (controlling for five personality traits) and frequency of writing words from the promotion and prevention categories. First, we observed a significant positive relationship between promotion (but not prevention) standards and using words from the promotion category. Second, the results revealed a significant positive relationship between prevention self-control and using words from the prevention category. Moreover, we observed a significant negative relationship between promotion self-control and using words from the prevention category. The full results are presented in S5 Table.

In line with pre-registration, we performed an additional sensitivity analysis. Namely, we excluded participants who did not use any word from both promotion and prevention categories from the analyses (i.e., had scores of 0 in both promotion and prevention results from LIWC output). The results were similar to results from the main analyses: a promotion focus was positively and significantly related to frequency of used words from the promotion category, and a prevention focus was positively and significantly related to frequency of used words from the prevention category. Furthermore, these additional analyses showed that a promotion focus was negatively and significant related to using words from the prevention category. Results are available in the S6 Table.

To summarize, we found support for both hypotheses. Results showed that a higher self-reported chronic promotion self-regulatory focus was related to a higher frequency of using words from the promotion category as well as a higher self-reported chronic prevention self-regulatory focus was related to a higher frequency of using words from the prevention category in the RF LIWC dictionary.

To corroborate our findings, we conducted an additional analysis on the texts provided by participants during Study 3. The writing samples (all 477 texts) were coded for their regulatory focus content by trained (Before texts rating, the competent judges participated in training. Namely, they had to read the literature about RF theory [88], complete a descriptive task where they described RF construct. Finally, they took part in an hour-long RF workshop.), independent research assistants (RAs) who were blind to the hypotheses (*N* = 7). The RAs rated each of the texts on both promotion and prevention using 5-point scales: from 1 [*definitely NOT promotion- / prevention-related]* to 5 (*definitely promotion- / prevention-related]*). ICC estimates (95% CI) were calculated using SPSS Statistics version 28 based on a mean-rating (*k* = 7), absolute-agreement, two-way mixed-effects model. The ICC for average measures was .82 (95% CI = .81–.84, p < .001), which means that the experts were similar in their ratings to a high extent [69]. Thus, judges’ ratings were averaged for promotion and prevention and these scores were correlated with both LIWC output as well as the results from the self-report questionnaire. Results of these correlational analyses are available in the S7 Table. As expected, we found a positive and statistically significant relationship between the frequency of words detected by LIWC from the promotion category in the writing samples and RA’s mean promotion ratings of these texts. Additionally, the results showed a positive and statistically significant relationship between the frequency of used words detected by LIWC from the prevention category in the writing sample and RA’s mean prevention ratings of these texts. Moreover, we found positive and statistically significant correlations between participants’ chronic promotion (but not prevention) self-regulation as measured by the self-report instrument and RA promotion ratings. Overall, the frequency with which particular words or word phrases are used as measured by LIWC were linked with more qualitative assessments by humans who were able to assess the responses with greater complexity.

## Discussion

The goal of this research was to adapt and validate a Polish version of the RF LIWC dictionary originally created by Gamache and colleagues [10]. This tool produces information about the number of promotion- and prevention-related words as a percentage of total words in the text. Below, we summarize the obtained findings and outline their implications for the literature.

First, we followed a thorough procedure to create the Polish equivalent of the original dictionary by (a) consulting three separate dictionaries, (b) seeking a consensus between two experts after independent choices for the entries were made, and (c) expanding the list by means of investigating Polish self-report instruments that capture RF. To establish the content validity of the adapted dictionary, we sought assistance from renowned RF experts from Poland, who rated the extent to which each word was related to each focus. The high agreement between the experts as well as a strong interaction effect between the source of the word (promotion vs. prevention category) and the experts’ ratings demonstrate the aptness of this instrument in capturing the two foci appropriately in the Polish language.

In the next step, we aimed to establish the dictionary’s divergent validity. An experimental manipulation aimed at inducing regulatory foci produced large differences between the groups of participants who were asked to describe their life from childhood till now: individuals in the promotion condition used more words from the promotion than from the prevention category, while the reverse was true for individuals in the prevention condition. The magnitude of these effects that appeared in the expected direction provides support for the discriminant validity of this instrument. Finally, we investigated whether the levels of promotion and prevention as measured via a traditional self-report questionnaire are related to the number of words from the promotion and prevention categories. This expectation was based on the assumptions made by Gamache and colleagues [10] that the RF dictionary can measure one’s chronic focus more implicitly. Controlling for personality traits, we demonstrated that the levels of promotion as measured via self-report explain the frequency of words from the promotion category in a writing sample: the higher the self-reported promotion, the more words used. The relationship was medium in size and promotion was the only meaningful predictor of the frequency of using words from the promotion category. We detected a similar pattern for prevention. The relationship between chronic prevention measured with a self-report questionnaire and the frequency of prevention words used in a writing passage was positive, significant, and medium in size. As expected, the Big Five personality traits were also not significantly linked with the frequency of prevention words and their relationship strength was even weaker. Interestingly, the relationship with chronic promotion was negative.

Overall, this line of research provided initial support for the validity of the Polish adaptation of the RF LIWC dictionary. The instrument has good content validity and allows meaningful differences in language to be detected as a result of activated RF. Thus, this instrument may be used as a relatively quick manipulation check after RF is manipulated and writing samples are obtained from participants. However, the results concerning LIWC as an implicit measure of chronic RF must be treated with caution. While the levels of promotion (prevention) focus as measured via questionnaire predicted a higher frequency of promotion (prevention) words beyond any other measured trait, the fit of the main model was unsatisfactory.

### Linguistic and cultural differences

In the process of validation of RF LIWC, one of the challenges we faced was related to differences in grammar between the original language (English) and the language for which translation was made (Polish). The English language is not governed by inflection to the same extent as Polish. For instance, the verb”broniłam” (ang.”I was defending”), derived from the infinitive “bronić”, covers information on the subject (it refers to the singular pronoun “I”) and its gender (feminine). It also refers to the imperfective past tense in an indicative mood. To make up for the fact that—compared to the English language—Polish verbs, nouns and adjectives have a larger number of forms, the Polish RF LIWC dictionary comprises a higher number of words in terms of their form, but a similar number in terms of the meaning (content) of these words. Additionally, because verbs in the Polish language contain information about the subject, the sentences do not formally require the operator, as the subject is inferred from the form of the verb. While that would in principal cause differences in LIWC results when it comes to the numbers of detected pronouns (as one of LIWC’s built-in categories), we believe that this feature of the Polish language should not affect RF LIWC scores.

The second important issue relates to the fact that in translations there are always differences caused by lack of strict correspondence between any two languages [89]. In each language the distribution of the meanings among words is slightly different; therefore, it is not possible to translate a text from one language to another without any differences. For example, the word “fail” has at least three equivalents in Polish: “błąd”, “porażka”, “mylić”. We minimized the differences between the two versions of RF LIWC by including distinct variants of the translated word to the Polish version of RF LIWC that were congruent with theory.

Similarly to Lazarević et al. [90], we believe that the mechanisms driving language production are not drastically different between English and Polish as two Indo-European languages. Thus, the patterns of results obtained with RF LIWC in Polish should be similar to those observed in English. As suggested by the works of Gamache and colleagues [13-online appendix], the examination of whether the English dictionary and its Polish adaptation are comparable in their performance should be part of the next important step in future validation processes. To provide evidence on cross-linguistic generalizability, the same texts written in Polish and English can be tested using RF LIWC in both language versions to observe their correspondence. For instance, corporate documents such as letters to shareholders written in both Polish and English can be compared, and the results for two LIWC outputs correlated. Another idea would be to compare texts written by bilingual (Polish-English) individuals.

### Limitations and future research

Our research has some limitations. First, our goal was to adapt an instrument that was previously validated and applied in several research projects from different fields, such as psychology (e.g., [12]), economics (e.g., [21]), management (e.g., [14]), or even energy policy (e.g., [91]). One could argue that a translation—regardless of the thoroughness of the procedure we applied—may not capture all relevant expressions concerning the foci in a specific language or cultural context. To limit this potential threat, we followed a procedure similar to Kanze et al. [55] where we analyzed the Polish instruments to measure regulatory foci [38,92] in order to identify relevant words/phrases that may have been absent from the list produced by Gamache and colleagues [10]. Some other scholars have also attempted to customize the dictionary and expand the original list [22]. Future developments in the Polish version of the RF LIWC dictionary could follow a similar path and extend the list. These attempts could include additional types of words that follow from RF theory. For instance, some studies have used LIWC’s standard dictionary categories to explore differences in the contents of participants’ descriptions of pursuing hopes and duties [93,94]. These studies have found that descriptions of duties contained more references to social processes and personal pronouns than descriptions of duties.

The next limitation is related to unsatisfactory fit of the main model (Model 1) in Study 3 (seven-factor model with two general dimensions: promotion and prevention foci). Since this model suffered from suboptimal fit [82], even though the modification indices were included, correlations of residuals of variables indicated by the calculation of modification indices were also included. This means that the results should be interpreted with caution. While a model that captures the division of each regulatory focus into standards and self-control was superior in terms of goodness of fit (see S4 Table) to a model with two general dimensions, two of four subscales of the PPSS scale had relatively low reliability (Cronbach’s *α* for promotion self-control = .65 and for prevention standards = .60). Thus, we suggest that another scale measuring chronic RF should be used in future studies.

Another limitation of our project is the fact that we did not report the internal consistency (Cronbach’s α) of word categories (see, e.g., Boyd et al., [95]. However, our linguistic analyses were based on the LIWC-2015 license, which does not provide the above-mentioned statistic. Reporting of the internal consistency of word categories seems to be a valid point for further validation studies (see [95]).

The next limitation relates to our study samples. In Studies 2 and 3 the participants were recruited from participant pools, and these individuals take part in studies to obtain university credit or monetary rewards. In study 2, this pool comprised students and it is possible that the results may not generalize beyond individuals with experience of higher education. It is possible that writing skills in this group are higher than in the general population, which may facilitate expressing oneself verbally to a higher extent. In study 2, the sample gender distribution was unequal (87% of women). This is a limitation, because some studies demonstrate links between gender and regulatory focus [96]. Study 3 comprised a wider population across the country, as indicated by the sample demographics. Yet, individuals who sign up to a research agency portal to become “professional participants” in scientific and marketing studies may also be a unique sample, differing from the general population. Therefore, future studies applying the Polish RF LIWC dictionary should be conducted among different populations, e.g., high school students, corporate employees, or pensioners. Another aspect that may limit the interpretation of our findings is related to the fact that writing samples provided online may differ from those provided in, e.g., laboratory settings [97]. It has been demonstrated that RF can be activated with contextual cues [98] and, therefore, the environment where one partakes in the study may affect what experiences are more salient and more likely to be described.

Our project consisted of three studies where the validity of the adaptation was tested by different means (Study 3). However, we did not test predictive validity, i.e., the extent to which this instrument allowed us to predict some unrelated, future outcomes. Future studies could test predictions about participants’ attitudes or behaviors, for example, decision making [43,99] or consumer behaviors [100] through measuring participants’ writing samples.

Finally, our pilot studies, which were performed in order to obtain a neutral instruction for Study 3, demonstrated that writing samples could be sensitive to different prompts. Future research could examine how well our findings generalize when differently worded writing prompts are used. Scholars aiming to measure individual RF with the RF LIWC dictionary should make sure that the instructions do not activate a specific focus, which may make it harder to detect vocabulary from the other focus, thus invalidating the method.

## Conclusions

In a series of studies, we adapted and provided preliminary validation for the Polish adaptation of the RF LIWC dictionary. Initial findings are promising. First, we show that Polish experts in RF can clearly and unanimously separate the wordlist into two categories. Second, prompted versus free writing samples demonstrate that activated versus chronic (respectively) RF is mirrored in the phrases used by the participants in their responses. These findings show that the dictionary may be used as a manipulation check after activating RF. Nevertheless, as the psychometric process is never truly over, we encourage future researchers to develop and validate this dictionary in their projects.

## Supporting information

S1 Table The final list of words for the RF LIWC Dictionary. Study 1. (DOCX)

S2 Table Statistics for pairwise comparisons between promotion and prevention ratings for each dictionary entry. Study 1. (DOCX)

S3 Table Regression parameters and statistics based on dataset with and without outliers. (DOCX)

S4 Table Goodness of fit indices of the structural equation models for Study 3. (DOCX)

S5 Table Additional SEM analysis in Study 3. (DOCX)

S6 Table Alternative SEM analyses. Study 3. (DOCX)

S7 Table Correlations between variables in Study 3 and competent judges’ ratings of texts’ promotion and prevention. (DOCX)

S1 Appendix Activation of promotion and prevention self-regulation. (DOCX)

S2 Appendix Versions of instructions. (DOCX)

S3 Appendix English translation of PPSS. (DOCX)

S4 Appendix Confirmatory Factor Analysis in Study 3. (DOCX)

## References

[1] Pennebaker JW, Mehl MR, Niederhoffer KG. Psychological Aspects of Natural Language Use: Our Words, Our Selves. Annu Rev Psychol. 2003;54: 547–577. doi: 10.1146/annurev.psych.54.101601.145041 12185209

[2] Tausczik YR, Pennebaker JW. The psychological meaning of words: LIWC and computerized text analysis methods. J Lang Soc Psychol. 2010;29: 24–54. doi: 10.1177/0261927X09351676

[3] Leis A, Ronzano F, Mayer MA, Furlong LI, Sanz F. Detecting signs of depression in tweets in Spanish: behavioral and linguistic analysis. J Med Internet Res. 2019;21: e14199. doi: 10.2196/14199 31250832 PMC6620890

[4] Alizadeh M, Weber I, Cioffi-Revilla C, Fortunato S, Macy M. Psychology and morality of political extremists: evidence from Twitter language analysis of alt-right and Antifa. EPJ Data Sci. 2019;8: 17. doi: 10.1140/epjds/s13688-019-0193-9

[5] Pietraszkiewicz A, Formanowicz M, Gustafsson Sendén M, Boyd RL, Sikström S, Sczesny S. The big two dictionaries: Capturing agency and communion in natural language. Eur J Soc Psychol. 2019;49: 871–887. doi: 10.1002/ejsp.2561

[6] Bazelli B, Hindle A, Stroulia E. On the personality traits of stackoverflow users. 2013 IEEE international conference on software maintenance. IEEE; 2013. pp. 460–463.

[7] Chung C, Pennebaker JW. The psychological functions of function words. Social Communication. New York, NY: Psychology Press; 2007. pp. 343–359.

[8] Pennebaker JW, King LA. Linguistic styles: language use as an individual difference. J Pers Soc Psychol. 1999;77: 1296–1312. doi: 10.1037//0022-3514.77.6.1296 10626371

[9] Zyung JD, Shi W. In retrospect: The influence of chief executive officers’ historical relative pay on overconfidence. Strateg Organ. 2022;20: 627–651. doi: 10.1177/14761270211004891

[10] Gamache DL, McNamara G, Mannor MJ, Johnson RE. Motivated to acquire? The impact of CEO regulatory focus on firm acquisitions. Acad Manage J. 2015;58: 1261–1282. doi: 10.5465/amj.2013.0377

[11] Higgins ET. Beyond pleasure and pain. Am Psychol. 1997;52: 1280–1300. doi: 10.1037//0003-066x.52.12.1280 9414606

[12] Conley MA, Higgins ET. Value from fit with distinct motivational field environments. Basic Appl Soc Psychol. 2018;40: 61–72. doi: 10.1080/01973533.2018.1434653

[13] Gamache DL, Neville F, Bundy J, Short CE. Serving differently: CEO regulatory focus and firm stakeholder strategy. Strateg Manag J. 2020;41: 1305–1335. doi: 10.1002/smj.3134

[14] Ma R, Hou W, Priem R, Wright P. Does restricted stock turn CEOs into risk-averse managers? Insights from the regulatory focus theory. Long Range Plann. 2022;55: 102165. doi: 10.1016/j.lrp.2021.102165

[15] Li A, Chiu SS, Kong D, Cropanzano R, Ho C-W. How CEOs respond to mortality salience During the COVID-19 pandemic: Integrating Terror Management Theory with Regulatory Focus Theory. J Appl Psychol. 2021;106: 1188–1201. doi: 10.1037/apl0000956 34424002

[16] Jancenelle VE. Tangible- Intangible resource composition and firm success. Technovation. 2021;108: 102337. doi: 10.1016/j.technovation.2021.102337

[17] Krippendorff K. Content analysis: An introduction to its methodology. Sage publications; 2018.

[18] Cycyota CS, Harrison DA. What (not) to expect when surveying executives: A meta-analysis of top manager response rates and techniques over time. Organ Res Methods. 2006;9: 133–160. doi: 10.1177/1094428105280770

[19] Hill AD, White MA, Wallace JC. Unobtrusive measurement of psychological constructs in organizational research. Organ Psychol Rev. 2014;4: 148–174. doi: 10.1177/2041386613505613

[20] Zhang Z, Gong M, Jia M. How and when does top management team regulatory focus influence firm environmental misconduct? Hum Relat. 2022;75: 1298–1326. doi: 10.1177/0018726721997531

[21] Gada VP, Popli M, Malhotra S. Time to complete the due diligence phase in mergers and acquisitions: impact of CEO psychological characteristics. Appl Econ. 2021;53: 5812–5825. doi: 10.1080/00036846.2021.1931005

[22] Jansen EJ, Miele DB, Fujita K, Scholer AA. Managing the motivation of others: Do managers recognize how to manage regulatory focus in subordinates? Motiv Sci. 2022;8: 330–345. doi: 10.1037/mot0000273

[23] Mount MP, Baer M. CEOs’ regulatory focus and risk-taking when firms perform below and above the bar. J Manag. 2022;48: 1980–2008. doi: 10.1177/01492063211016029

[24] Duriau VJ, Reger RK, Pfarrer MD. A content analysis of the content analysis literature in organization studies: Research themes, data sources, and methodological refinements. Organ Res Methods. 2007;10: 5–34. doi: 10.1177/1094428106289252

[25] Ahmadi S, Khanagha S, Berchicci L, Jansen JJ. Are managers motivated to explore in the face of a new technological change? The role of regulatory focus, fit, and complexity of decision-making. J Manag Stud. 2017;54: 209–237. doi: 10.1111/joms.12257

[26] Kanze D, Huang L, Conley MA, Higgins ET. We ask men to win and women not to lose: Closing the gender gap in startup funding. Acad Manage J. 2018;61: 586–614. doi: 10.5465/amj.2016.1215

[27] Malhotra S, Reus TH, Zhu P, Roelofsen EM. The acquisitive nature of extraverted CEOs. Adm Sci Q. 2018;63: 370–408. doi: 10.1177/0001839217712240

[28] Scoresby RB, Withers MC, Ireland RD. The effect of CEO regulatory focus on changes to investments in R&D. J Prod Innov Manag. 2021;38: 401–420. doi: 10.1111/jpim.12591

[29] Freitas AL, Higgins ET. Enjoying Goal-Directed Action: The Role of Regulatory Fit. Psychol Sci. 2002;13: 1–6. doi: 10.1111/1467-9280.00401 11892772

[30] Higgins ET. Promotion and prevention: regulatory focus as a motivational principle. Adv Exp Soc Psychol. 1998;30: 1–46. doi: 10.1016/S0065-2601(08)60381-0

[31] Kahneman D, Tversky A. On the interpretation of intuitive probability: A reply to Jonathan Cohen. Cognition. 1979; 409–411. doi: 10.1016/0010-0277(79)90024-6

[32] Higgins ET, Cornwell JFM. Securing foundations and advancing frontiers: Prevention and promotion effects on judgment & decision making. Organ Behav Hum Decis Process. 2016;136: 56–67. doi: 10.1016/j.obhdp.2016.04.005

[33] Higgins ET, Liberman N. The loss of loss aversion: Paying attention to reference points. J Consum Psychol. 2018;28: 523–532. doi: 10.1002/jcpy.1045

[34] Lanaj K, Chang C-H “Daisy”, Johnson RE. Regulatory focus and work-related outcomes: A review and meta-analysis. Psychol Bull. 2012;138: 998–1034. doi: 10.1037/a0027723 22468880

[35] Johnson PD, Smith MB, Wallace JC, Hill AD, Baron RA. A review of multilevel regulatory focus in organizations. J Manag. 2015;41: 1501–1529. doi: 10.1177/0149206315575552

[36] Zou X, Scholer AA, Higgins ET. Risk preference: How decision maker’s goal, current value state, and choice set work together. Psychol Rev. 2020;127: 74–94. doi: 10.1037/rev0000162 31414876

[37] Higgins ET. Self-discrepancy: a theory relating self and affect. Psychol Rev. 1987;94: 319–340. doi: 10.1037/0033-295X.94.3.319 3615707

[38] Kolańczyk A, Bak W, Roczniewska M. Skala samoregulacji promocyjnej i prewencyjnej (SSPP). Psychol Spoleczna. 2013;8: 203–218.

[39] Higgins ET, Roney CJR, Crowe E, Hymes C. Ideal versus ought predilections for approach and avoidance distinct self-regulatory systems. J Pers Soc Psychol. 1994;66: 276–286. doi: 10.1037//0022-3514.66.2.276 8195986

[40] Higgins ET. Making a good decision: Value from fit. Am Psychol. 2000;55: 1217–1230. doi: 10.1037/0003-066X.55.11.1217 11280936

[41] Higgins ET. How self-regulation creates distinct values: the case of promotion and prevention decision making. J Consum Psychol. 2002;12: 177–191. doi: 10.1207/S15327663JCP1203_01

[42] Scholer AA, Higgins ET. Distinguishing levels of approach and avoidance: An analysis using regulatory focus theory. Handbook of approach and avoidance motivation. New York, NY: Psychology Press; 2008. pp. 489–503.

[43] Crowe E, Higgins ET. Regulatory focus and strategic inclinations: promotion and prevention in decision-making. Organ Behav Hum Decis Process. 1997;69: 117–132. doi: 10.1006/obhd.1996.2675

[44] Förster J, Higgins ET, Bianco AT. Speed/accuracy decisions in task performance: Built-in trade-off or separate strategic concerns? Organ Behav Hum Decis Process. 2003;90: 148–164. doi: 10.1016/S0749-5978(02)00509-5

[45] Hamstra MRW, Bolderdijk JW, Veldstra JL. Everyday risk taking as a function of regulatory focus. J Res Personal. 2011;45: 134–137. doi: 10.1016/j.jrp.2010.11.017

[46] Idson LC, Higgins ET. How current feedback and chronic effectiveness influence motivation: Everything to gain versus everything to lose. Eur J Soc Psychol. 2000;30: 583–592.

[47] Van-Dijk D, Kluger AN. Feedback sign effect on motivation: Is it moderated by regulatory focus? Appl Psychol. 2004;53: 113–135. doi: 10.1111/j.1464-0597.2004.00163.x

[48] Higgins ET, Friedman RS, Harlow RE, Idson LC, Ayduk ON, Taylor A. Achievement orientations from subjective histories of success: Promotion pride versus prevention pride. Eur J Soc Psychol. 2001;31: 3–23. doi: 10.1002/ejsp.27

[49] Fellner B, Holler M, Kirchler E, Schabmann A. Regulatory Focus Scale (RFS): Development of a scale to record dispositional regulatory focus. Swiss J Psychol. 2007;66: 109–116. doi: 10.1024/1421-0185.66.2.109

[50] Lockwood P, Jordan CH, Kunda Z. Motivation by positive or negative role models: regulatory focus determines who will best inspire us. J Pers Soc Psychol. 2002;83: 854–864. doi: 10.1037/0022-3514.83.4.854 12374440

[51] Higgins ET, Shah J, Friedman R. Emotional responses to goal attainment: strength of regulatory focus as moderator. J Pers Soc Psychol. 1997;72: 515–525. doi: 10.1037//0022-3514.72.3.515 9120782

[52] Paulhus DL, Vazire S. The self-report method. Handbook of research methods in personality psychology. Guilford; 2007. pp. 224–239.

[53] Podsakoff PM, MacKenzie SB, Lee J-Y, Podsakoff NP. Common method biases in behavioral research: a critical review of the literature and recommended remedies. J Appl Psychol. 2003;88: 879–903. doi: 10.1037/0021-9010.88.5.879 14516251

[54] Pan L, McNamara G, Lee JJ, Haleblian J (John), Devers CE. Give it to us straight (most of the time): Top managers’ use of concrete language and its effect on investor reactions. Strateg Manag J. 2018;39: 2204–2225. doi: 10.1002/smj.2733

[55] Kanze D, Conley MA, Higgins ET. The motivation of mission statements: How regulatory mode influences workplace discrimination. Organ Behav Hum Decis Process. 2021;166: 84–103. doi: 10.1016/j.obhdp.2019.04.002

[56] Pham T, Mathmann F, Jin HS, Higgins ET. How regulatory focus–mode fit impacts variety‐seeking. J Consum Psychol. 2023;33: 77–96. doi: 10.1002/jcpy.1317

[57] Gamache DL, McNamara G. Responding to bad press: How CEO temporal focus influences the sensitivity to negative media coverage of acquisitions. Acad Manage J. 2019;62: 918–943. doi: 10.5465/amj.2017.0526

[58] Graf-Vlachy L, Bundy J, Hambrick DC. Effects of an advancing tenure on CEO cognitive complexity. Organ Sci. 2020;31: 936–959. doi: 10.1287/orsc.2019.1336

[59] Short JC, McKenny AF, Reid SW. More than words? Computer-aided text analysis in organizational behavior and psychology research. Annu Rev Organ Psychol Organ Behav. 2018;5: 415–435. doi: 10.1146/annurev-orgpsych-032117-104622

[60] Gaur A, Kumar M. A systematic approach to conducting review studies: An assessment of content analysis in 25 years of IB research. J World Bus. 2018;53: 280–289. doi: 10.1016/j.jwb.2017.11.003

[61] Jiang W, Wang L, Chu Z, Zheng C. How Does CEO Regulatory Focus Matter? The Impacts of CEO Promotion and Prevention Focus on Firm Strategic Change. Group Organ Manag. 2020;45: 386–416. doi: 10.1177/1059601119891268

[62] Johnsen J-AK, Vambheim SM, Wynn R, Wangberg SC. Language of motivation and emotion in an internet support group for smoking cessation: Explorative use of automated content analysis to measure regulatory focus. 2014 [cited 8 Oct 2022]. doi: 10.2147/PRBM.S54947 24470780 PMC3896322

[63] Pandey A, Tripathi S. To go or to let it go: A regulatory focus perspective on Bundle Consumption. J Serv Res. 2023;26: 136–150. doi: 10.1177/10946705211067101

[64] Cambridge Dictionary | English Dictionary, Translations & Thesaurus. [cited 13 Nov 2022]. https://dictionary.cambridge.org/.

[65] Słownik PONS | Definicje, Tłumaczenia, Słownictwo. [cited 14 Nov 2022]. https://pl.pons.com/t%C5%82umaczenie.

[66] Słowniki online bab.la—loving languages. [cited 13 Nov 2022]. //pl.bab.la/.

[67] wsjppan. Wielki słownik języka polskiego PAN. In: wsjp.pl [Internet]. wsjppan; [cited 13 Nov 2022]. https://wsjp.pl.

[68] Słownik języka polskiego PWN. [cited 13 Nov 2022]. https://sjp.pwn.pl/.

[69] Koo TK, Li MY. A guideline of selecting and reporting intraclass correlation coefficients for reliability research. J Chiropr Med. 2016;15: 155–163. doi: 10.1016/j.jcm.2016.02.012 27330520 PMC4913118

[70] Nunnally B, Bernstein IR. Psychometric Theory. 3rd ed. New York: Oxford Univer. Press.; 1994.

[71] Faul F, Erdfelder E, Lang A-G, Buchner A. G*Power 3: A flexible statistical power analysis program for the social, behavioral, and biomedical sciences. Behav Res Methods. 2007;39: 175–191. doi: 10.3758/bf03193146 17695343

[72] Morris SB, DeShon RP. Combining effect size estimates in meta-analysis with repeated measures and independent-groups designs. Psychol Methods. 2002;7: 105–125. doi: 10.1037/1082-989x.7.1.105 11928886

[73] Cesario J, Higgins ETT, Scholer AA. Regulatory fit and persuasion: basic principles and remaining questions. Soc Personal Psychol Compass. 2008;2: 444–463. doi: 10.1111/j.1751-9004.2007.00055.x

[74] Hoyle RH. Personality and self-regulation. Handbook of personality and self-regulation. Blackwell Publishing Ltd; 2010. pp. 1–18.

[75] Barrick MR, Stewart GL, Piotrowski M. Personality and job performance: Test of the mediating effects of motivation among sales representatives. J Appl Psychol. 2002;87: 43–51. doi: 10.1037/0021-9010.87.1.43 11916215

[76] Topolewska E, Skimina E, Strus W, Cieciuch J, Rowiński T. Krótki kwestionariusz do pomiaru Wielkiej Piątki IPIP-BFM-20. Ann Psychol. 2014;17: 367–384.

[77] The lavaan Project. [cited 14 Nov 2022]. https://lavaan.ugent.be/tutorial/.

[78] Rosseel Y, Oberski D, Byrnes J, Vanbrabant L, Savalei V, Merkle E, et al. Package ‘lavaan.’ June. 2017;17: 1.

[79] Rosseel Y. lavaan: An R package for structural equation modeling. J Stat Softw. 2012;48: 1–36. doi: 10.18637/jss.v048.i02

[80] R: The R project for statistical computing. [cited 14 Nov 2022]. https://www.r-project.org/.

[81] Browne MW, Cudeck R. Alternative ways of assessing model fit. Sociol Methods Res. 1992;21: 230–258. doi: 10.1177/0049124192021002005

[82] Hu L, Bentler PM. Cutoff criteria for fit indexes in covariance structure analysis: Conventional criteria versus new alternatives. Struct Equ Model Multidiscip J. 1999;6: 1–55. doi: 10.1080/10705519909540118

[83] Maydeu-Olivares A, Shi D. Correction to Maydeu-Olivares & Shi, 2017: (10.1017/1614-2241/a000129). Methodology. 2017;13: 157–157. doi: 10.1017/1614-2241/a000129

[84] Whittaker TA. Using the modification index and standardized expected parameter change for model modification. J Exp Educ. 2012;80: 26–44. doi: 10.1080/00220973.2010.531299

[85] Preacher KJ, Coffman DL. Computing power and minimum sample size for RMSEA [Computer software]. http://quantpsy.org/. 2006.

[86] Sim M, Kim S-Y, Suh Y. Sample size requirements for simple and complex mediation models. Educ Psychol Meas. 2022;82: 76–106. doi: 10.1177/00131644211003261 34992307 PMC8725051

[87] Kock N, Lynn G. Lateral collinearity and misleading results in variance-based SEM: An illustration and recommendations. J Assoc Inf Syst. 2012;13: 546–580. doi: 10.17705/1jais.00302

[88] Bąk W, Łaguna M, Bondyra-Łuczka E. Kwestionariuszowe metody pomiaru ukierunkowań regulacyjnych. Polskie adaptacje kwestionariuszy RFQ i RF. Psychol Społeczna. 2015; 84–99. doi: 10.7366/1896180020153206

[89] Szymczyk B, Zakowicz W, Stemplewska-Zakowicz K. Automatyczna analiza tekstu: polska adaptacja programu LIWC Jamesa Pennebakera. Przegląd Psychol. 2012;55: 195–209.

[90] Lazarević LB, Bjekić J, Živanović M, Knežević G. Ambulatory assessment of language use: Evidence on the temporal stability of Electronically Activated Recorder and stream of consciousness data. Behav Res Methods. 2020;52: 1817–1835. doi: 10.3758/s13428-020-01361-z 32016918

[91] Endrejat PC, Güntner AV, Ehrenholz S, Kauffeld S. Tailored communication increases the perceived benefits of solar energy. Energy Policy. 2020;144: 111714. doi: 10.1016/j.enpol.2020.111714

[92] Roczniewska M, Retowski S, Osowiecka M, Słomska I, Wrońska M. Work Regulatory Focus scale—Polish adaptation. Pol J Appl Psychol. 2013;12: 115–136.

[93] Vaughn LA. Contents of Hopes and Duties: A Linguistic Analysis. Front Psychol. 2018;9: 757. doi: 10.3389/fpsyg.2018.00757 29867701 PMC5964360

[94] Vaughn LA. Distinguishing Between Need Support and Regulatory Focus with LIWC. Vazire S, Corker K, editors. Collabra Psychol. 2019;5: 32. doi: 10.1525/collabra.185

[95] Boyd RL, Ashokkumar A, Seraj S, Pennebaker JW. The development and psychometric properties of LIWC-22. Austin TX Univ Tex Austin. 2022; 1–47.

[96] Wang L, Cui Y, Wang X, Wang J, Du K, Luo Z. Regulatory Focus, Motivation, and Their Relationship With Creativity Among Adolescents. Front Psychol. 2021;12: 666071. doi: 10.3389/fpsyg.2021.666071 34093361 PMC8172616

[97] Burkins P. The language of regulatory focus: Words used in descriptions of different promotion- and prevention-focused experiences. PsyArXiv; 2021. doi: 10.31234/osf.io/tg2sh

[98] Higgins ET. Beyond pleasure and pain: How motivation works. 1st edition. Oxford; New York: Oxford University Press; 2013.

[99] Werth L, Foerster J. How regulatory focus influences consumer behavior. Eur J Soc Psychol. 2007;37: 33–51. doi: 10.1002/ejsp.343

[100] Higgins ET, Nakkawita E, Cornwell JFM. Beyond outcomes: How regulatory focus motivates consumer goal pursuit processes. Consum Psychol Rev. 2020;3: 76–90. doi: 10.1002/arcp.1052

