# Supplementary material for: Promotion and prevention regulatory focus LIWC dictionary. Polish adaptation and validation
Source: PLoS One. 2023 Jul 20;18(7):e0288726. doi: 10.1371/journal.pone.0288726 (PMC10358899; doi:10.1371/journal.pone.0288726)
Supplement: S1 Table — (DOCX) [file pone.0288726.s001.docx]

| S1 Table. The final list of words for the RF LIWC Dictionary. Study 1. | | | | |
| --- | --- | --- | --- | --- |
|  | Table 1a | | Table 1b | |
|  | Words from the promotion category | | Words from the prevention category | |
| **No** | **RF (Promotion) Words (Gamache, 2015)** | **Polish (promotion) Words Evaluated by Experts in Study 1** | **RF (Prevention) Words (Gamache, 2015)** | **Polish (prevention) Words Evaluated by Experts in Study 1** |
| 1 | accomplish | osiągać* | accuracy | poprawność* |
| 2 | achieve | spełnienie* | afraid | obawa* |
| 3 | advancement | rozwój* | anxious | niepokój* |
| 4 | aspiration | dążyć* | avoid | ustrzec* |
| 5 | aspire | aspirować* | careful | ostrożność, sprawdzać* |
| 6 | attain | zdobywać* | conservative | zachowawczy* |
| 7 | desire | pragnienie*, marzenie* | defend | bronić* |
| 8 | earn | zarabiać | duty | obowiązek* |
| 12 | expand | zwiększać* | escape | umykać* |
| 13 | gain | korzyść* | escaping | NONE |
| 14 | grow | dojrzewać* | evade | unikać* |
| 15 | hope | nadzieja* | fail | błąd*, porażka*, mylić* |
| 16 | hoping | NONE | fear | lęk* |
| 17 | ideal | ideał* | loss | strata* |
| 18 | improve | ulepszać*, doskonalić* | obligation | NONE |
| 19 | increase | rosnąć* | ought | powinność* |
| 20 | momentum | pęd* | pain | przykrość* |
| 21 | obtain | zysk* | prevent | zapobiegać* |
| 22 | optimistic | optymizm* | protect | chronić* |
| 23 | progress | postęp* | responsible | odpowiedzialność* |
| 24 | promoting | promować* | risk | ryzyko |
| 25 | promotion | wspierać* | safety | bezpieczeństwo* |
| 26 | speed | spieszyć* | security | NONE |
| 27 | swift | szybkość* | threat | zagrożony* |
| 28 | toward | w kierunku* | vigilance | czujność*, uważność |
| 29 | velocity | prędkość* | --- | --- |
| 30 | wish | chcieć* | --- | --- |
| 31 | NONE | wyzwanie* | --- | --- |
|  | |  |  |  |
| Note. Words with "*" are contained in the final list of RF LIWC | | | |  |
