# Supplementary material for: Promotion and prevention regulatory focus LIWC dictionary. Polish adaptation and validation
Source: PLoS One. 2023 Jul 20;18(7):e0288726. doi: 10.1371/journal.pone.0288726 (PMC10358899; doi:10.1371/journal.pone.0288726)
Supplement: S2 Table — (DOCX) [file pone.0288726.s002.docx]

| S2 Table. Statistics for pairwise comparisons between promotion and prevention ratings for each dictionary entry. Study 1. | | | | | | | | | | | | | | | | | | | |  |  |
| --- | --- | --- | --- | --- | --- | --- | --- | --- | --- | --- | --- | --- | --- | --- | --- | --- | --- | --- | --- | --- | --- |
| S2 Table A | | | | | | | | | | | S2 Table B | | | | | | | | | | |
| RF (Promotion) Words (Gamache, 2015) | Polish (promotion) words evaluated by experts in Study 1 | Mean for "promotion" words - rating as promotion | SD Mean - rating as promotion | Mean for "promotion" words - rating as prevention | SD Mean - rating as prevention | t test | p value | Cohen's d \| Paired Samples Effect Sizes | Lower CI | Upper CI | RF (Prevention) Words (Gamache, 2015) | Polish (prevention) words evaluated by experts in Study 1 | Mean for "prevention" words - rating as promotion | SD Mean - rating as promotion | Mean for "prevention" words - rating as prevention | SD Mean - rating as prevention | t test | p value | Cohen's d \| Paired Samples Effect Sizes | Lower CI | Upper CI |
| aspire | aspirować* | 4.90 | 0.32 | 1.30 | 0.68 | 16.28 | < .001 | 5.15 | 2.73 | 7.56 | safety | bezpieczeństwo* | 1.40 | 0.70 | 4.90 | 0.32 | -13.02 | < .001 | -4.12 | -6.08 | -2.14 |
| wish | chcieć | 4.20 | 0.63 | 2.30 | 0.95 | 4.15 | .002 | 1.31 | 0.43 | 2.15 | fail | błąd* | 1.80 | 0.79 | 4.20 | 1.03 | -4.61 | .001 | -1.46 | -2.35 | -0.53 |
| aspiration | dążyć* | 4.60 | 0.52 | 1.80 | 0.79 | 8.57 | < .001 | 2.71 | 1.32 | 4.08 | defend | bronić* | 1.30 | 0.48 | 4.70 | 0.48 | -12.75 | < .001 | -4.03 | -5.95 | -2.09 |
| grow | dojrzewać* | 3.40 | 0.84 | 2.20 | 0.92 | 3.34 | .009 | 1.06 | 0.26 | 1.82 | protect | chronić* | 1.40 | 0.70 | 4.80 | 0.42 | -11.13 | < .001 | -3.52 | -5.22 | -1.80 |
| NONE | doskonalić* | 4.40 | 0.84 | 1.80 | 0.79 | 5.46 | < .001 | 1.73 | 0.71 | 2.71 | vigilance | czujność* | 2.10 | 1.20 | 4.10 | 1.20 | -2.74 | .023 | -0.87 | -1.58 | -0.12 |
| ideal | ideał* | 4.60 | 0.70 | 1.80 | 1.03 | 6.00 | < .001 | 1.90 | 0.82 | 2.94 | fear | lęk* | 1.40 | 0.70 | 4.80 | 0.42 | -12.75 | < .001 | -4.03 | -5.95 | -2.09 |
| gain | korzyść* | 4.10 | 0.88 | 2.30 | 0.95 | 3.38 | .008 | 1.07 | 0.26 | 1.84 | NONE | mylić* | 1.90 | 0.74 | 3.80 | 0.79 | -4.67 | .001 | -1.48 | -2.37 | -0.55 |
| NONE | marzenie* | 4.60 | 0.70 | 1.60 | 0.84 | 7.61 | < .001 | 2.41 | 1.14 | 3.65 | anxious | niepokój* | 1.80 | 0.79 | 4.60 | 0.52 | -7.20 | < .001 | -2.28 | -3.47 | -1.06 |
| hope | nadzieja* | 4.20 | 0.63 | 1.80 | 0.92 | 6.47 | < .001 | 2.05 | 0.91 | 3.15 | escaping | NONE | NONE | NONE | NONE | NONE | NONE | NONE | NONE | NONE | NONE |
| hoping | NONE | NONE | NONE | NONE | NONE | NONE | NONE | NONE | NONE | NONE | security | NONE | NONE | NONE | NONE | NONE | NONE | NONE | NONE | NONE | NONE |
| optimistic | optymizm* | 4.60 | 0.52 | 1.70 | 0.68 | 10.47 | < .001 | 3.31 | 1.68 | 4.93 | obligation | NONE | NONE | NONE | NONE | NONE | NONE | NONE | NONE | NONE | NONE |
| accomplish | osiągać* | 4.70 | 0.48 | 2.10 | 0.88 | 8.51 | < .001 | 2.69 | 1.31 | 4.05 | afraid | obawa* | 1.60 | 0.84 | 4.60 | 0.52 | -7.61 | < .001 | -2.41 | -3.65 | -1.14 |
| momentum | pęd* | 4.00 | 0.67 | 2.10 | 0.99 | 4.15 | .002 | 1.31 | 0.43 | 2.15 | duty | obowiązek* | 1.70 | 0.82 | 4.60 | 0.70 | -7.13 | < .001 | -2.25 | -3.44 | -1.04 |
| progress | postęp* | 4.50 | 0.71 | 1.70 | 1.06 | 5.25 | .001 | 1.66 | 0.67 | 2.62 | responsible | odpowiedzialność* | 2.30 | 1.06 | 4.20 | 0.79 | -3.61 | .006 | -1.14 | -1.93 | -0.32 |
| desire | pragnienie* | 4.70 | 0.48 | 2.10 | 1.10 | 6.09 | < .001 | 1.93 | 0.84 | 2.98 | careful | ostrożność* | 1.30 | 0.48 | 4.70 | 0.48 | -12.75 | < .001 | -4.03 | -5.95 | -2.09 |
| velocity | prędkość* | 3.20 | 0.92 | 2.30 | 0.68 | 2.86 | .019 | 0.91 | 0.14 | 1.63 | accuracy | poprawność* | 1.80 | 0.63 | 4.40 | 0.70 | -7.01 | < .001 | -2.22 | -3.38 | -1.02 |
| promoting | promować* | 4.60 | 0.70 | 1.50 | 0.85 | 6.43 | < .001 | 2.03 | 0.91 | 3.13 | NONE | porażka* | 1.50 | 0.71 | 4.40 | 0.84 | -6.33 | < .001 | -2.00 | -3.09 | -0.89 |
| increase | rosnąć* | 4.40 | 0.70 | 1.90 | 0.57 | 6.71 | < .001 | 2.12 | 0.96 | 3.25 | ought | powinność* | 1.60 | 0.84 | 4.70 | 0.48 | -8.19 | < .001 | -2.59 | -3.90 | -1.25 |
| advancement | rozwój* | 4.70 | 0.48 | 1.50 | 0.71 | 8.91 | < .001 | 2.82 | 1.38 | 4.23 | pain | przykrość* | 1.90 | 0.99 | 3.80 | 1.03 | -6.04 | < .001 | -1.91 | -2.96 | -0.83 |
| achieve | spełnienie* | 4.10 | 0.88 | 2.10 | 0.88 | 4.47 | .002 | 1.41 | 0.50 | 2.29 | risk | ryzyko | 2.70 | 1.70 | 3.40 | 1.78 | -0.65 | .531 | -0.21 | -0.83 | 0.43 |
| speed | spieszyć | 2.70 | 0.82 | 2.40 | 1.08 | 0.82 | .434 | 0.26 | -0.38 | 0.88 | NONE | sprawdzać* | 2.00 | 0.67 | 4.40 | 0.52 | -9.00 | < .001 | -2.85 | -4.27 | -1.40 |
| swift | szybkość* | 3.40 | 0.70 | 2.20 | 0.79 | 4.13 | .003 | 1.31 | 0.43 | 2.15 | loss | strata* | 1.30 | 0.68 | 4.60 | 0.70 | -7.80 | < .001 | -2.47 | -3.73 | -1.17 |
| improve | ulepszać* | 4.40 | 0.70 | 2.10 | 1.20 | 5.81 | < .001 | 1.84 | 0.78 | 2.86 | escape | umykać* | 1.90 | 0.99 | 3.80 | 1.03 | -3.48 | .007 | -1.10 | -1.88 | -0.29 |
| toward | w kierunku* | 3.60 | 0.70 | 2.40 | 0.70 | 3.34 | .009 | 1.06 | 0.26 | 1.82 | evade | unikać* | 1.20 | 0.42 | 4.90 | 0.32 | -24.22 | < .001 | -7.66 | -11.19 | -4.13 |
| promotion | wspierać* | 3.50 | 0.53 | 2.50 | 0.85 | 2.74 | .023 | 0.87 | 0.12 | 1.58 | avoid | ustrzec* | 1.40 | 0.52 | 4.80 | 0.42 | -15.38 | < .001 | -4.86 | -7.15 | -2.57 |
| NONE | wyzwanie* | 4.70 | 0.68 | 1.60 | 0.84 | 7.15 | < .001 | 2.26 | 1.05 | 3.45 | NONE | uważność | 2.80 | 1.14 | 3.70 | 0.82 | -1.59 | .147 | -0.50 | -1.15 | 0.17 |
| earn | zarabiać | 3.30 | 0.82 | 2.40 | 0.97 | 1.96 | .081 | 0.62 | -0.07 | 1.29 | conservative | zachowawczy* | 1.10 | 0.32 | 5.00 | 0.00 | -39.00 | < .001 | -12.33 | -17.96 | -6.72 |
| attain | zdobywać* | 4.80 | 0.42 | 1.40 | 0.52 | 12.75 | < .001 | 4.03 | 2.09 | 5.95 | threat | zagrożony* | 1.50 | 0.53 | 4.60 | 0.52 | -9.86 | < .001 | -3.12 | -4.65 | -1.56 |
| expand | zwiększać* | 4.20 | 0.42 | 1.90 | 0.74 | 6.87 | < .001 | 2.17 | 0.99 | 3.32 | prevent | zapobiegać* | 1.50 | 0.71 | 4.80 | 0.42 | -11.00 | < .001 | -3.48 | -5.16 | -1.77 |
| obtain | zysk* | 4.50 | 0.70 | 1.60 | 0.84 | 6.02 | < .001 | 1.90 | 0.82 | 2.95 | - | - | - | - | - | - | - | - | - | - | - |
|  |  |  |  |  |  |  |  |  |  |  |  |  |  |  |  |  |  |  |  |  |  |
| Note. Words with "*" are contained in the final list of RF LIWC | | | | | | | |  |  |  |  |  |  |  |  |  |  |  |  |  |  |
