# Supplementary material for: Promotion and prevention regulatory focus LIWC dictionary. Polish adaptation and validation
Source: PLoS One. 2023 Jul 20;18(7):e0288726. doi: 10.1371/journal.pone.0288726 (PMC10358899; doi:10.1371/journal.pone.0288726)
Supplement: S3 Table — (DOCX) [file pone.0288726.s003.docx]

| S3 Table. Regression parameters and statistics based on dataset with and without outliers. Based on Cook's distance - 3 times mean of Cook's distances.  Models with Log frequency using words from the promotion category and prevention category as dependent variables. Study 3 | | | | | | | | | | |
| --- | --- | --- | --- | --- | --- | --- | --- | --- | --- | --- |
|  |  |  |  |  |  |  |  |  |  |  |
|  | Dataset 1 - with outliers (DV - promotion words) | Dataset 1a - without ouliers (DV - promotion words) | Dataset 2 - with outliers (DV - prevention words) | Dataset 2a - without outliers (DV - prevention words) |  |  |  |  |  |  |
| Adj. R-squared | .01 | .05 | .01 | .03 |  |  |  |  |  |  |
| p-value | .099 | < .001 | .146 | .016 |  |  |  |  |  |  |
|  |  |  |  |  |  |  |  |  |  |  |
| Note. Dataset 1 - with outliers (DV - dependent variable: promotion words): Regression parameters and statistics based on dataset with outliers (based on Cook's distance - 3 times mean of Cook's distances); N = 477 | | | | | | | | | | |
| Dataset 1a - without outliers (DV - dependent variable: promotion words): Regression parameters and statistics based on dataset without outliers (based on Cook's distance - 3 times mean of Cook's distances); N = 443 | | | | | | | | | | |
| Dataset 2 - with outliers (DV - dependent variable: prevention words): Regression parameters and statistics based on dataset with outliers (based on Cook's distance - 3 times mean of Cook's distances); N = 477 | | | | | | | | | | |
| Dataset 2a - without outliers (DV - dependent variable: prevention words): Regression parameters and statistics based on dataset without outliers (based on Cook's distance - 3 times mean of Cook's distances); N = 442 | | | | | | | | | | |
