# Supplementary material for: Promotion and prevention regulatory focus LIWC dictionary. Polish adaptation and validation
Source: PLoS One. 2023 Jul 20;18(7):e0288726. doi: 10.1371/journal.pone.0288726 (PMC10358899; doi:10.1371/journal.pone.0288726)
Supplement: S4 Table — (DOCX) [file pone.0288726.s004.docx]

| S4 Table. Goodness of fit indices of the structural equation models for Study 3 | | | | | | | | | |
| --- | --- | --- | --- | --- | --- | --- | --- | --- | --- |
|  |  |  |  |  |  |  |  |  |  |
| Model | χ2 | df | CFI | Robust CFI | TLI | Robust TLI | RMSEA | Robust RMSEA | SRMR |
| Model 1 | 1933 | 778 | .81 | .82 | .79 | .80 | .06 | .06 | .09 |
| Model 2 | 2197 | 785 | .76 | .77 | .74 | .75 | .07 | .06 | .10 |
| Model 3a | 1006 | 400 | .86 | .87 | .84 | .84 | .06 | .06 | .07 |
| Model 3b | 1186 | 461 | .84 | .85 | .82 | .83 | .06 | .06 | .08 |
|  |  |  |  |  |  |  |  |  |  |
| Note. *N* = 414. |  |  |  |  |  |  |  |  |  |
| Model 1 = seven-factor model - including Big Five Personality traits, promotion and prevention foci. Modification indices employed. | | | | | | | | | |
| Model 2 = seven-factor model - including Big Five Personality traits, promotion and prevention foci. Without modification indices. | | | | | | | | | |
| Model 3a = seven-factor model - including Big Five Personality traits, promotion standards and prevention standards. | | | | | | | | | |
| Model 3b = seven-factor model - including Big Five Personality traits, promotion control and prevention control. | | | | | | | | | |
