# Supplementary material for: Promotion and prevention regulatory focus LIWC dictionary. Polish adaptation and validation
Source: PLoS One. 2023 Jul 20;18(7):e0288726. doi: 10.1371/journal.pone.0288726 (PMC10358899; doi:10.1371/journal.pone.0288726)
Supplement: S5 Table — (DOCX) [file pone.0288726.s005.docx]

| S5 Table. Additional SEM analysis in Study 3.  Promotion and prevention self-regulation are additionally divided into standards and self-control factors. | | | | | | | | | | | |
| --- | --- | --- | --- | --- | --- | --- | --- | --- | --- | --- | --- |
|  |  |  | |  |  |  |  |  |  |  |  |
|  |  | | two-factors self-regulations - model for promotion standards and prevention standards + five personality traits | | | | two-factors self-regulations - model for promotion self-control and prevention self-control + five personality traits | | | |  |
| Dependent variable | predictor | | Estimate (*b*) | *SE* | *p* | *β* | *Estimate (b)* | *SE* | *p* | *β* |  |
| Frequency of words from promotion category after log- transformation | Promotion self-regulation: standards | | **0.31** | **0.09** | **< .001** | **.29** | ---- | ---- | ---- | ---- |  |
|  | Promotion self-regulation: self-control | | ---- | ---- | ---- | ---- | 0.06 | 0.10 | .583 | .08 |  |
|  | Prevention self-regulation: standards | | 0.14 | 0.08 | .094 | .13 | ---- | ---- | ---- | ---- |  |
|  | Prevention self-regulation: self-control | | ---- | ---- | ---- | ---- | 0.14 | 0.11 | .206 | .10 |  |
|  | Extraversion | | -0.02 | 0.04 | .578 | -.04 | -0.02 | 0.05 | .689 | -.03 |  |
|  | Agreeableness | | 0.11 | 0.10 | .255 | .07 | 0.11 | 0.10 | .252 | .07 |  |
|  | Conscientiousness | | **-0.11** | **0.05** | **.021** | **-.12** | -0.11 | 0.06 | .099 | -.13 |  |
|  | Neuroticism | | -0.08 | 0.05 | .122 | -.13 | -0.09 | 0.05 | .067 | -.14 |  |
|  | Intellect | | -0.05 | 0.06 | .452 | -.06 | -0.01 | 0.07 | .932 | -.01 |  |
| Frequency of words from prevention category after log- transformation | Promotion self-regulation: standards | | -0.10 | 0.06 | .105 | -.13 | ---- | ---- | ---- | ---- |  |
|  | Promotion self-regulation: self-control | | ---- | ---- | ---- | ---- | **-0.21** | **0.10** | **.035** | **-.40** |  |
|  | Prevention self-regulation: standards | | -0.02 | 0.06 | .776 | -.02 | ---- | ---- | ---- | ---- |  |
|  | Prevention self-regulation: self-control | | ---- | ---- | ---- | ---- | **0.26** | **0.10** | **.008** | **.27** |  |
|  | Extraversion | | -0.01 | 0.03 | .800 | -.02 | 0.04 | 0.03 | .250 | .11 |  |
|  | Agreeableness | | 0.01 | 0.07 | .936 | .01 | -0.03 | 0.08 | .714 | -.02 |  |
|  | Conscientiousness | | -0.01 | 0.04 | .747 | .-02 | **-0.11** | **0.05** | **.041** | **-.18** |  |
|  | Neuroticism | | -0.01 | 0.04 | .812 | -.02 | -0.06 | 0.04 | .111 | -.13 |  |
|  | Intellect | | 0.05 | 0.05 | .307 | .09 | 0.08 | 0.07 | .249 | .13 |  |
| Note. *N* = 414. |  |  | |  |  |  |  |  |  |  |  |
