# Supplementary material for: Promotion and prevention regulatory focus LIWC dictionary. Polish adaptation and validation
Source: PLoS One. 2023 Jul 20;18(7):e0288726. doi: 10.1371/journal.pone.0288726 (PMC10358899; doi:10.1371/journal.pone.0288726)
Supplement: S6 Table — (DOCX) [file pone.0288726.s006.docx]

| S6 Table. Alternative SEM analyses. Study 3.  The analyses after excluding participants with 0 scores in promotion and prevention RF LIWC output in Study 3.  Two-factors regulatory focus (promotion and prevention) with the modification based on modification indices + five personality traits. | | | | | |
| --- | --- | --- | --- | --- | --- |
|  |  |  | | | |
| Dependent variable | predictor | *Estimate (b)* | *SE* | *p* | *β* |
| Frequency of words from promotion category after log-transformation | Promotion self-regulation | **0.33** | **0.11** | **.003** | **.39** |
|  | Prevention self-regulation | -0.11 | 0.17 | .518 | -.06 |
|  | Extraversion | -0.03 | 0.04 | .518 | -.05 |
|  | Agreeableness | 0.01 | 0.10 | .951 | .01 |
|  | Conscientiousness | 0.04 | 0.06 | .497 | .06 |
|  | Neuroticism | -0.01 | 0.05 | .869 | -.01 |
|  | Intellect | -0.10 | 0.08 | .202 | -.14 |
| Frequency of words from prevention category after log-transformation | Promotion self-regulation | **-0.30** | **0.10** | **.004** | **-.41** |
|  | Prevention self-regulation | **0.42** | **0.20** | **.041** | **.28** |
|  | Extraversion | 0.03 | 0.04 | .467 | .06 |
|  | Agreeableness | -0.06 | 0.10 | .544 | -.06 |
|  | Conscientiousness | 0.01 | 0.05 | .874 | .02 |
|  | Neuroticism | -0.02 | 0.05 | .635 | -.05 |
|  | Intellect | 0.09 | 0.06 | .172 | .15 |
| Note. *N* = 352 |  |  |  |  |  |
