# Supplementary material for: Promotion and prevention regulatory focus LIWC dictionary. Polish adaptation and validation
Source: PLoS One. 2023 Jul 20;18(7):e0288726. doi: 10.1371/journal.pone.0288726 (PMC10358899; doi:10.1371/journal.pone.0288726)
Supplement: S7 Table — (DOCX) [file pone.0288726.s007.docx]

| S7 Table. Correlations between variables in Study 3 and competent judges ratings of texts' promotion and prevention | | | | | | | | |
| --- | --- | --- | --- | --- | --- | --- | --- | --- |
|  |  |  |  |  |  |  |  |  |
|  | Prom_judg | Prew_judg | Prom_chron_partic | Prev_chron_partic | LogPromLIWC |  |  |  |
| Prom_judg | 1 |  |  |  |  |  |  |  |
| N | 477 |  |  |  |  |  |  |  |
| Prew_judg | -.75** | 1 |  |  |  |  |  |  |
| N | 477 | 477 |  |  |  |  |  |  |
| Prom_chron_partic | .28** | -.15** | 1 |  |  |  |  |  |
| N | 414 | 414 | 414 |  |  |  |  |  |
| Prev_chron_partic | -.04 | .09 | .11* | 1 |  |  |  |  |
| N | 414 | 414 | 414 | 414 |  |  |  |  |
| LogPromLIWC | .20** | -.11* | .22** | .06 | 1 |  |  |  |
| N | 414 | 414 | 414 | 414 | 414 |  |  |  |
| LogPrevLIWC | -.16** | .19** | -.08 | .10 | .06 |  |  |  |
|  | 414 | 414 | 414 | 414 | 414 |  |  |  |
| Note. Prom_judg—promotion-related rating by competent judges; Prew_judg—prevention-related rating by competent judges; Prom_chron_partic—chronic promotion-related assessment by participant in Study 3; Prev_chron_partic—chronic prevention-related assessment by participant in Study; LogPromLIWC - Log. transformated frequency of used "promotion" words in Study 3; LogPrevLIWC - Log. transforamted frequency of used "prevention" words in Study 3 | | | | | | | |  |
| ** Correlation is significant at the 0.01 level (2-tailed). | | | | | |  |  |  |
| * Correlation is significant at the 0.05 level (2-tailed). | | | | | |  |  |  |
