# Supplementary material for: Promotion and prevention regulatory focus LIWC dictionary. Polish adaptation and validation
Source: PLoS One. 2023 Jul 20;18(7):e0288726. doi: 10.1371/journal.pone.0288726 (PMC10358899; doi:10.1371/journal.pone.0288726)
Supplement: S1 Appendix — (DOCX) [file pone.0288726.s008.docx]

# **S1 Appendix**

**Activation of promotion and prevention self-regulation**

**Activation of promotion self-regulation (English – translated version).**

For the purposes of this study, please think about how your current **hopes** and **aspirations** differ from those you had when you were growing up? In other words, what **achievements** would you like to have at this stage of your life? What **achievements** did you want to achieve when you were a child?

In the box below, write a short story about how your **hopes and aspirations** have changed over time: from when you were a child to now.

Your text should consist of a minimum of 2100 characters with spaces, i.e., about 300 words, i.e., about half a page - it is very important for research purposes that the story is NOT too short.

**Activation of prevention self-regulation (English – translated version).**

For the purposes of this study, please think about how your current **duties and responsibilities** differ from those you had when you were growing up? In other words, what **responsibilities** should you be fulfilling at this stage of your life? What **responsibilities** should you have fulfilled when you were a child?

In the box below, write a short story about how your **duties** and **responsibilities** have changed over time: from when you were a child until now.

Your text should consist of a minimum of 2100 characters with spaces, i.e., about 300 words, i.e., about half a page - it is very important for research purposes that the story is NOT too short.

**Activation of promotion self-regulation (Polish – original version).**

Na potrzeby tego badania pomyśl proszę o tym, czym różnią się Twoje obecne **nadzieje i aspiracje** od tych, które miałeś(-aś), gdy dorastałeś(-aś)? Innymi słowy, jakie **osiągnięcia** chciałbyś/chciałabyś mieć na obecnym etapie swojego życia? Jakie **osiągnięcia** chciałeś(-aś) mieć, gdy byłeś(-aś) dzieckiem?

W poniższym polu napisz krótkie opowiadanie, przedstawiające to, jak zmieniały się Twoje **nadzieje i aspiracje** na przestrzeni czasu: od wtedy, gdy byłeś(-aś) dzieckiem, aż do teraz.

Twój tekst powinien składać się z minimum 2100 znaków ze spacjami, czyli około 300 słów, tj. około pół strony – bardzo ważne dla celów badawczych jest to, żeby opowiadanie NIE było zbyt krótkie.

**Activation of prevention self-regulation (Polish – original version)**

Na potrzeby tego badania pomyśl proszę o tym, czym różnią się Twoje obecne **powinności i obowiązki** od tych, które miałeś(-aś), gdy dorastałeś(-aś)? Innymi słowy, jakie **obowiązki** powinieneś/powinnaś wypełniać na obecnym etapie swojego życia? Jakie **obowiązki** powinieneś był wypełniać / powinnaś była wypełniać, gdy byłeś(-aś) dzieckiem?

W poniższym polu napisz krótkie opowiadanie, przedstawiające to, jak zmieniały się Twoje **powinności i obowiązki** na przestrzeni czasu: od wtedy, gdy byłeś(-aś) dzieckiem, aż do teraz.

Twój tekst powinien składać się z minimum 2100 znaków ze spacjami, czyli około 300 słów, tj. około pół strony – bardzo ważne dla celów badawczych jest to, żeby opowiadanie NIE było zbyt krótkie.
