# Supplementary material for: Promotion and prevention regulatory focus LIWC dictionary. Polish adaptation and validation
Source: PLoS One. 2023 Jul 20;18(7):e0288726. doi: 10.1371/journal.pone.0288726 (PMC10358899; doi:10.1371/journal.pone.0288726)
Supplement: S2 Appendix — (DOCX) [file pone.0288726.s009.docx]

# **S2 Appendix**

**Versions of instructions**

**English version of instructions (translated)**

Pilot Study 1: Think about and describe in the box below what education, career or personal life issues/matters are most important to you right now? How are you going to address them?

Pilot Study 2: Think about various current topics in your life. Describe in the box below what is important to you now and how you are paying attention to it?

Pilot Study 3: Think about various current goals in your life. Describe in the box below what is important to you right now and how you are paying attention to it?

**Polish version of instructions (original)**

Pilot Study 1: Zastanów się i opisz w polu poniżej, jakie kwestie / sprawy dotyczące edukacji, kariery zawodowej lub życia prywatnego są dla Ciebie teraz najważniejsze? W jaki sposób zamierzasz się nimi zająć?

Pilot Study 2: Zastanów się nad różnymi aktualnymi tematami w Twoim życiu. Opisz w polu poniżej, co jest teraz dla Ciebie ważne i jak poświęcasz temu uwagę?

Pilot Study 3: Zastanów się nad różnymi aktualnymi celami w Twoim życiu. Opisz w polu poniżej, co jest teraz dla Ciebie ważne i jak poświęcasz temu uwagę?
