# Supplementary material for: Promotion and prevention regulatory focus LIWC dictionary. Polish adaptation and validation
Source: PLoS One. 2023 Jul 20;18(7):e0288726. doi: 10.1371/journal.pone.0288726 (PMC10358899; doi:10.1371/journal.pone.0288726)
Supplement: S3 Appendix — (DOCX) [file pone.0288726.s010.docx]

# **S3 Appendix**

**English translation of PPSS**

Note. The translation of PPSS presented in this Appendix was prepared only for the purpose of this article. English version has not been validated.

**English version of PPSS (translated)**

Below you will find statements about yourself. Read them and indicate to what extent they characterize you. According to the given scale, 1 means: I strongly disagree, and 5: I strongly agree. There are no right or wrong answers here – please be honest with your answers.

1 – strongly disagree

2 – disagree

3 – neither agree or disagree

4 – agree

5 – strongly agree

1. I like taking up new challenges.

2. When performing a task, I focus on making as few mistakes as possible.

3. When I want something, ideas of “how to get it” come easily to me.

4. I often pay special attention to what I do so that it is not perceived negatively by others.

5. Fulfilling my own aspirations and continual development are the most important things in my life.

6. In life, I usually do what I want to do.

7. When I have a task to do, I spend all the time necessary to do the task to do it to the best of my ability.

8. I give up easily.

9. I often think about what I should do and what I shouldn't do.

10. I’m governed by the rule – “where there’s a will, there’s a way”.

11. Most often, my actions are consistent with what others expect from me.

12. I usually do what I have to do.

13. It’s difficult to get discouraged when I make up my mind about something.

14. I like to act spontaneously.

15. I’m not discouraged by minor failures when I’m striving for something important.

16. I often think about potential problems when I’m working on a task.

17. I usually create an alternative plan, a so-called ‘plan B’.

18. I go for my dreams.

19. While performing a task, I usually look for something that gives me joy.

20. I try very hard to stick to the chosen course of action and not give up.

21. When I start a task, I make sure I understand the instructions correctly.

22. I'm pretty persistent.

23. I act and I turn dreams into reality.

24. Before I do anything, I carefully assess my abilities.

25. I know that I can be wrong and that’s why I act cautiously.

26. I often give up on a previously-set goal.

27. I consider my own dreams more important than doing what others expect from me.

Subscales: promotion standards (items: 5, 6,18, 23, 27), promotion self-control (items: 1, 3, 14, 19), prevention standards (items: 4, 9, 11, 12), prevention n self-control (items: 2, 7, 16, 17, 21, 24, 25), strength of motivation (items: 8, 10, 13, 15, 20, 22, 26).

**Polish version of PPSS (original)**

Poniżej znajdują się twierdzenia na temat samego siebie. Zapoznaj się z nimi i wskaż, w jakim stopniu one Cię charakteryzują? Zgodnie z podaną skalą 1 oznacza: całkowicie się nie zgadzam, a 5: w pełni się zgadzam. Nie ma tu dobrych lub złych odpowiedzi – prosimy odpowiadać szczerze.

1 – całkowicie nie zgadzam się

2 – nie zgadzam się

3 – trochę się zgadzam, trochę się nie zgadzam

4 – zgadzam się

5 – w pełni zgadzam się

1. Lubię podejmować nowe wyzwania.

2. Wykonując zadanie, koncentruję się na tym, aby popełnić jak najmniej błędów.

3. Jeżeli czegoś chcę, to pomysły „jak to zrobić” łatwo przychodzą mi do głowy.

4. Często kieruję się tym, aby to, co robię, nie było odebrane źle przez innych.

5. Spełnianie własnych aspiracji i ciągły rozwój są najistotniejsze w moim życiu.

6. W życiu na ogół robię to, co chcę.

7. Kiedy mam jakieś zadanie, cały czas sprawdzam, czy je dobrze rozumiem i rozwiązuję.

8. Łatwo się poddaję.

9. Często myślę nad tym, co powinnam/powinienem, a czego nie powinnam/nie powinienem robić.

10. Kieruję się zasadą – „chcieć to móc”.

11. Moje działania najczęściej są zgodne z tym, czego oczekują ode mnie inni.

12. Robię zwykle to, co muszę.

13. Trudno mnie zniechęcić, gdy już coś postanowię.

14. Lubię działać spontanicznie.

15. Nie zniechęcają mnie drobne porażki, gdy robię coś ważnego.

16. Często zastanawiam się nad problemami, które mogą zaistnieć w czasie mojego działania.

17. Zazwyczaj opracowuję plan awaryjny (tzw. plan B).

18. Marzę i spełniam swoje marzenia.

19. W zadaniu zwykle szukam czegoś, co sprawia mi przyjemność.

20. Bardzo staram się w życiu trzymać obrany kierunek działania i nie poddawać się.

21. Kiedy zaczynam wykonywać zadanie, upewniam się, czy dobrze rozumiem jego instrukcję.

22. W porównaniu z innymi ludźmi jestem wytrwały/-a.

23. Działając, marzenia zmieniam w rzeczywistość.

24. Zanim się za coś wezmę, uważnie oceniam swoje możliwości.

25. Wiem, że mogę się mylić, dlatego działam ostrożnie.

26. Często zdarza mi się rezygnować z raz obranego celu.

27. Ważniejsza jest dla mnie realizacja moich marzeń niż wykonywanie tego, czego oczekują ode mnie inni.

Podskale: standardy promocyjne (itemy: 5, 6, 18, 23, 27), kontrola promocyjna (itemy: 1, 3, 14, 19), standardy prewencyjne (itemy: 4, 9, 11, 12), kontrola prewencyjna (itemy:2, 7, 16, 17, 21, 24, 25), siła motywacji (itemy: 8, 10, 13, 15, 20, 22, 26).
