# Supplementary material for: Promotion and prevention regulatory focus LIWC dictionary. Polish adaptation and validation
Source: PLoS One. 2023 Jul 20;18(7):e0288726. doi: 10.1371/journal.pone.0288726 (PMC10358899; doi:10.1371/journal.pone.0288726)
Supplement: S4 Appendix — (DOCX) [file pone.0288726.s011.docx]

# **S4 Appendix**

**Confirmatory Factor Analysis in Study 3**

To test the construct validity of the measures used in Study 3, we conducted a series of confirmatory factor analyses (CFA) using the lavaan package [1,2] in R software [3] . The proposed model assumed seven factors: promotion self-regulation, prevention self-regulation, extraversion, agreeableness, conscientiousness, neuroticism and intellect.

This model was compared to two alternative structures by testing the change in χ2 values: (a) a one-factor model with all items loading onto one factor and (b) a two-factor model with regulatory focus (promotion and prevention foci as one factor) and The Big Five Personality Traits (all dimensions as one factor). In line with the multifaceted approach to assessment of model fit, we considered the following fit indices: Comparative Fit Index (CFI [4]) Tucker and Lewis Index (TLI [5]), Root Mean Square Error of Approximation (RMSEA [6]), along with 90 % confidence interval (CI) limits, and (Standardized) Root Mean Square Residual ([S]RMR [4]). We used the following values as thresholds recommended in the literature: TLI and CFI > .90 [4,5], RMSEA < .08 [6], and (S)RMR < .08 [4] .

We tested the multinormality assumptions and observed that it was violated (significant multivariate skew and kurtosis tests). Additionally, Likert scales, which we applied in Study 3 could be considered ordinal data, especially if the number of response categories is lower than 10 [7]. In that case, maximum likelihood estimation with robust standard errors (MLR) method could be applied to test model fit. Therefore, below we also present fit values for robust estimation.

The results indicated that the proposed 7-factor model (promotion self-regulation, prevention self-regulation, extraversion, agreeableness, conscientiousness, neuroticism and intellect) fitted the data reasonably well: *χ^2^* = 2104; df = 719, RMSEA / Robust RMSEA = .07 / .07 [90% CI: .06―.07]; CFI / Robust CFI = .77 / .78; TLI / Robust TLI = .75 / .76; SRMR = .10. As the indices indicate, while RMSEA scores were acceptable, CFI.TLI scores were below the acceptable thresholds. Yet, this measurement model was superior (*Δχ^2^* = 1080, Δdf = 20, *p* < .001) to a two-factor model (regulatory focus, Big Five Personality traits): *χ^2^* = 3827, *df* = 739, RMSEA / Robust RMSEA = .10 / .10 [90% CI: .10―.10], CFI / Robust CFI = .48 / .48, TLI / Robust TLI= .45 / .46, SRMR = .11. This two-factor model also fit the data better (*Δχ^2^* = 59, *Δdf* = 1, *p* < .001) than a one-factor model χ^2^ = 4080, df = 740, RMSEA / Robust RMSEA = .10 / .10 [90% CI: .10―.11], CFI / Robust CFI = .43 / .44, TLI / Robust TLI = .40 / .41, SRMR = .11.

Overall, the proposed structure with seven factors is superior to other solutions. However, due to unsatisfactory CFI/TLI scores, it should be analyzed with caution.

# References

1. Rosseel Y. lavaan: An R package for structural equation modeling. J Stat Softw. 2012;48: 1–36. doi:10.18637/jss.v048.i02

2. The lavaan Project. [cited 14 Nov 2022]. Available: https://lavaan.ugent.be/tutorial/

3. R: The R project for statistical computing. [cited 14 Nov 2022]. Available: https://www.r-project.org/

4. Hu L, Bentler PM. Cutoff criteria for fit indexes in covariance structure analysis: Conventional criteria versus new alternatives. Struct Equ Model Multidiscip J. 1999;6: 1–55. doi:10.1080/10705519909540118

5. Bagozzi RP, Yi Y. On the evaluation of structural equation models. J Acad Mark Sci. 1988;16: 74–94. doi:10.1007/BF02723327

6. Browne MW, Cudeck R. Alternative ways of assessing model fit. Sociol Methods Res. 1992;21: 230–258. doi:10.1177/0049124192021002005

7. Wu H, Leung S-O. Can Likert Scales be Treated as Interval Scales?—A Simulation Study. J Soc Serv Res. 2017;43: 527–532. doi:10.1080/01488376.2017.1329775
